# Circ_0000181 regulates miR-667-5p/NLRC4 axis to promote pyroptosis progression in diabetic nephropathy

**DOI:** 10.1038/s41598-022-15607-7

**Published:** 2022-07-14

**Authors:** Yining Li, Weihong Yu, Hao Xiong, Fang Yuan

**Affiliations:** 1 Center of Organ Transplantation, The Second Xiangya Hospital of Central South University, Changsha, 410011 China; 2 Department of Nephrology, The Second Xiangya Hospital of Central South University, No 139 Renmin Road, Changsha, 410011 China

**Keywords:** Biochemistry, Proteins

## Abstract

Our previous research demonstrated that NOD-like receptor family CARD domain-containing protein 4 (NLRC4) inflammasome was overexpressed in renal tissues of patients with diabetic nephropathy (DN). This study further investigated the effect of circRNAs-miRNAs interaction on NLRC4 and their potential mechanisms. DN mice models were first established using STZ. Then, pyroptosis related marker expression was detected using qPCR, western blot (WB), and immunohistochemistry analysis. After that, differentially expressed circRNAs, miRNAs, and mRNAs were investigated using next-generation sequencing. Additionally, the function and potential mechanism of circ_0000181 and miR-667-5p on pyroptosis were measured in vitro DN cell model using MTS, WB, and Enzyme-linked immunosorbent assay. There was an apparent elevation of NLRC4, Caspase1, IL-1β, and IL-18 levels in DN mice. The next-generation sequencing results revealed that there were 947 circRNAs and 390 miRNAs significantly different between the DN and sham kidney tissue, of which circ_0000181 and miR-667-5p had potential targeting effects with NLRC4. Dual-luciferase and functional rescue experiments demonstrated that circ_0000181 promoted NLRC4 inflammasome activation via competitive sponge of miR-667-5p, promoted the release of IL-1β and IL-18, and caused pyroptosis. Altogether, circ_0000181 regulates miR-667-5p/NLRC4 axis to promote pyroptosis progression in DN.

## Introduction

Diabetic nephropathy (DN), an important complication of patients with diabetes, has become a major cause of end-stage renal disease^1^. DN mainly manifests as increased proteinuria, albumin excretion, and reduced glomerular filtration rate, which has a significant effect on patients’ quality of life and economic burden. However, the mechanism of DN remains unclarified. Pro-inflammatory cytokines have been suggested to be a factor that contributes to DN. As DN begins, macrophages and T cells accumulation is found in the kidney, and inflammatory factors, such as TNF-α, IL-1, and IL-6, are released^2^. It has been reported that chronic hyperglycemia can induce macrophage accumulation and activation, leading to the deposition of glomerular immune complexes, and the production of inflammatory factors to promote DN aggravation^3^. Additionlly, inflammasomes, such as NLRP3 and NLRC4, have been shown to be involved in DN development^4,5^.

NLRC4 is an inflammasome that is complexly composed of pattern recognition receptors and significantly influences insulin resistance and diabetes. Induced by exogenous pathogen-related or risk-related molecular patterns (PAMPs/DAMPs) stimulation^6,7^, inflammasomes are activated and proteolytically activate pro-interleukin 1 family cytokines, IL-1β and IL-18, triggering inflammatory cell death^8^. Among them, activated inflammasome NLRC4 directly interacts with Caspase1 and recruitment domain protein that activates Caspase1^9^. Moreover, by indirectly sensing bacterial flagellin and the type III secretion system, inflammasome complexes are formed to promote macrophage Caspase1 activation and pyrolysis^10^. As an immune complex, NLRC4 plays a vital role in inflammation factors’ activation^11^. It has been shown that NLRC4 inflammasome activation regulates the regulation of tumors and autoimmune diseases^7,12^. Notably, NLRC4 activation has also been confirmed to be involved in regulating DN progression^5^. Therefore, a better understanding of NLRC4’s role and function in the DN pathogenesis may lead to the development of new treatment strategies.

Circular RNA (circRNA) is a kind of non-coding formed by splicing the upstream splicing donor site and the adopter site downstream of RNA transcription through an end-to-end connection during transcription. CircRNA have 5’ to 3’ polarity and does not contain a polymer-adenylated tail^13^. circRNA acts as a microRNA (miRNA) sponge to antagonize the interaction between miRNA and its target mRNA. Evidence show that circRNA is involved in regulating DN progress. CircRNA_15698 and circular RNA HIPK3 promote DNA progress by regulating the miR-185 pathway^14,15^. Circ_0000491 regulates the accumulation of glomerular mesangial cells extracellular matrix through miR-101b/TGFβ pathway and promotes DN progression^16^. These results suggest that circRNA is involved in DN regulation. However, whether circRNA regulates DN through NLRC4 activation remains to be reported.

This study aims to investigate the possible mechanism by which circRNA regulates DN by activating NLRC4. By establishing an animal model of DN, we confirmed that NLRC4 expression in the kidney of DN mice was significantly increased, thereby activating Caspase1’s enzyme activity. Additionally, through next-generation sequencing analysis, we demonstrated that circ_0000181 mediated NLRC4 expression by regulating miR-667-5p, promoting the release of IL-1β and IL-18 from renal tubular epithelial cells, and causing pyrolysis.

## Results

### The pathological characteristics and NLRC4 expression in the DN model

**Figure 1:**
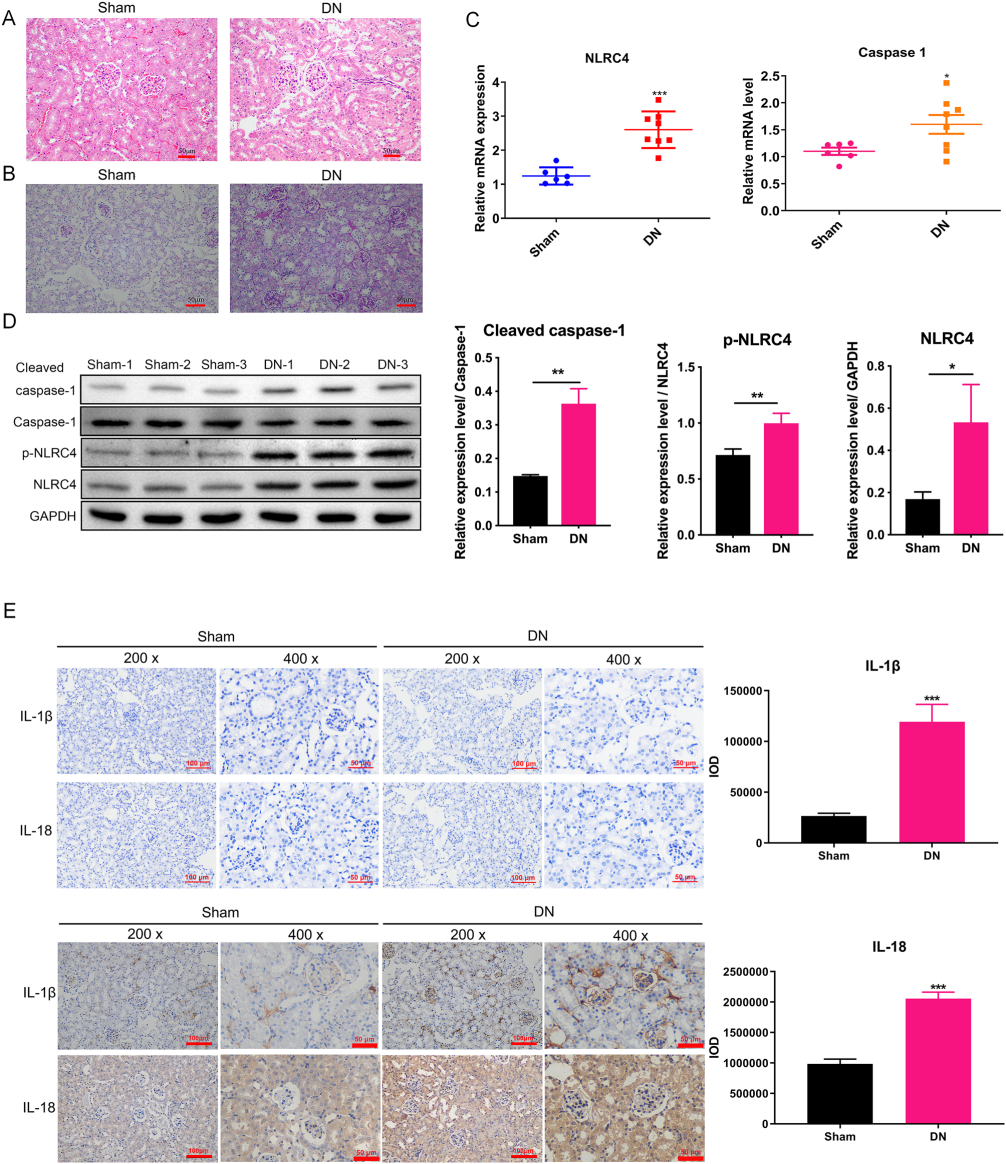
The pathological characteristics and NLRC4 expression in DN mice. (**A**) The H&E staining of the kidney tissues was used to show the changes in structure of the sham (n = 3) and DN mice (n = 3). (**B**) The PAS staining of the kidney tissues was used to show the content of sugars in sham (n = 3) and DN mice (n = 3). (**C**) The mRNA levels of NLRC4 and Caspase1 were measured using qPCR in the kidney tissues of sham (n = 6) and DN mice (n = 8). (**D**) The WB analysis was used to detect NLRC4 and Caspase1 protein levels in the kidney tissues of sham (n = 3) and DN mice (n = 3). The origianl images were cut prior to hybridisation with antibodies during blotting. (**E**) The IHC analysis was used to determine the expression of IL-1β and IL-18 in the kidney tissues of sham (n = 3) and DN mice (n = 3). Upper left panel is negative controls for IHC with no primary antibodies incubation, lower left panel is the results using IL-1β and IL-18 antibody incubation. Right is the quantification of the lower left panel, and IOD equal to mean density ( average reaction intensity of positive cells) multiply by area of positive cells. *P < 0.05; **P < 0.01; ***P < 0.001. Data are presented as the means ± SD from three independent repetitions.

H&E and PAS staining showed that in the DN model, the kidney pathology was obvious, the glomerular basement membrane was thickened, the extracellular and mesangial matrices were more accumulated, the renal tubule thickness was decreased, and more sugars were accumulated (Fig. 1A,B). The above phenomena could show successful DN model construction. We further detected NLRC4 and Caspase1 expressions in the DN model, and the results confirmed significant NLRC4 and Caspase1 upregulation in mRNAs (Fig. 1C). and WB results also revealed that the protein levels of Cleaved Caspase1, p-NLRC4, and NLRC4 were also elevated in the DN group relative to that in the sham group (Fig. 1D). The inflammatory factors (IL-1β and IL-18) were also apparently upregulated in the DN model via immunohistochemical analysis (Fig. 1E). Altogether, these data strongly suggested that NLRC4 was significantly increased in DN mice, which activated Caspase1 and promoted IL-1β and IL-18 expressions.

### The bioinformatics analysis of differential expressed circRNAs, miRNAs, and mRNAs in the DN model

**Figure 2:**
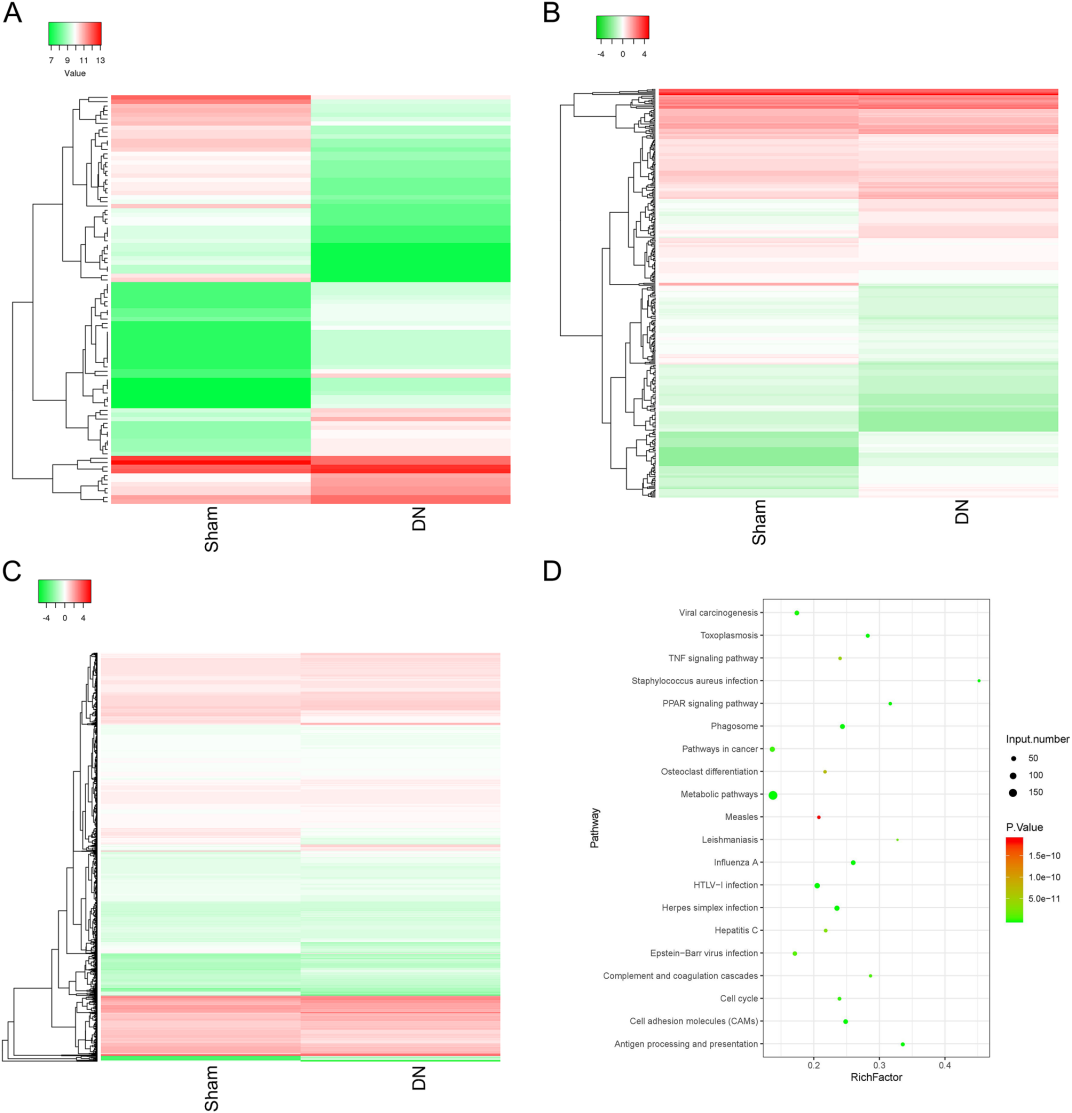
The bioinformatics analysis of differentially expressed circRNAs, miRNAs, and mRNAs between the DN and sham groups. (**A–C**) The clustering plot was used to show the differentially expressed circRNAs (**A**), miRNAs (**B**), and mRNAs (**C**). (**D**) Differentially expressed mRNAs were used for KEGG analysis.

To accurately locate DN influence on circRNAs, miRNAs, and mRNAs levels, next-generation sequencing analysis was performed on the whole genome of kidney tissue in the DN and sham groups. The hierarchical clustering analysis of differentially expressed circRNAs, miRNAs, and mRNAs between the two groups is presented in Fig. 2A–C. Additionally, 947 differentially expressed circRNAs were identified: 515 upregulated and 432 downregulated circRNAs (Table S1). There were 390 differentially expressed miRNAs identified, including 128 upregulated and 262 downregulated miRNAs (Table S2). Also, 2992 differentially expressed mRNAs were identified: 2373 upregulated and 619 downregulated mRNAs in the DN mice versus the sham mice (Table S3). Then, KEGG analysis was conducted to investigate the functions of differentially expressed mRNAs and identify signaling pathways that respond to DN. The results showed that the primary signaling pathways of differentially expressed mRNAs were the metabolic pathway, TNF signaling pathway, and cell cycle as well as CAMs pathway (Fig. 2D).

### The selection of targeted circRNAs and miRNAs to the NLRC4 gene in the DN model

**Figure 3:**
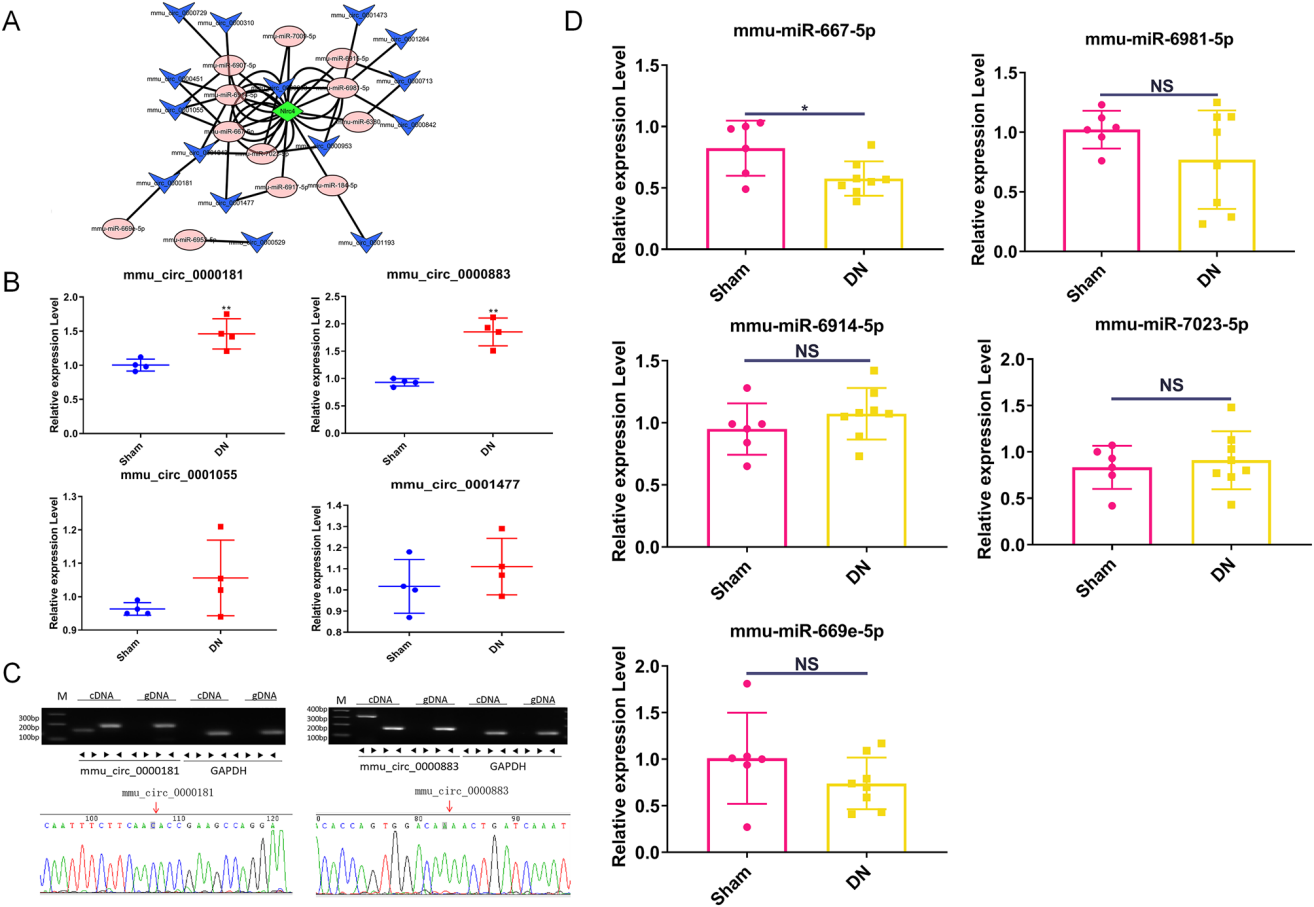
The selection of targeted circRNAs and miRNAs to the NLRC4 gene in the DN model. (**A**) The circRNAs-miRNAs-NLRC4 network was plotted using cytoscape software. (**B**) qPCR analysis was used to detect circRNAs expression in the kidney tissue of sham (n = 4) and DN mice (n = 4). (**C**) The circular structures of circ_0000181 and circ_0000883 were validated via agarose gel electrophoresis and Sanger sequencing. The original images of agarose gel electrophoresis were got ignore the edges visible. (**D**) The expression of miRNAs was detected using qPCR in the kidney tissue of sham (n = 6) and DN mice (n = 8). *P < 0.05; **P < 0.01. Data are presented as the means ± SD from three independent repetitions.

We established a network regulation map of circRNAs and miRNAs associated with NLRC4 (Fig. 3A and Table S4) based on a previous study that reported the parent gene of circRNAs involved in kidney diseases to further investigate the potential regulated mechanism of NLRC4^17–20^. Additionally, the screening conditions are the length of circRNA between 350 and 3000-bp and the number of circRNA and miRNA binding sites > 1. Furthermore, we screened the circRNA, having a significance difference in expression, and the parent gene is related to the kidney. Based on these two conditions, we found that four circRNAs (circ_0000181, circ_0000883, circ_0001055, and circ_0001477) and five miRNAs (miR-667-5p, miR-6981-5p, miR-6914-5p, miR-7023-5p, and miR-699e-5p) (Table S5). Additionally, the expression of multiple changes of the four circRNAs and five miRNAs are shown in Fig. S1. Then four circRNAs were verified using qPCR in the sequencing samples, and the results showed that the circRNAs with significantly different expressions were circ_0000181 and circ_0000883 (Fig. 3B). Additionally, the authenticity of their circular structures was further confirmed using agarose gel electrophoresis and Sanger sequencing (Fig. 3C). Furthermore, five miRNAs expressions were also detected using qPCR, and only miR-667-5p expression had a significant difference between the sham and DN groups (Fig. 3D). Thus, circ_0000181, circ_0000883, and miR-667-5p, were selected for further study.

### circ_0000181 is involved in the pyroptosis mediated by NLRC4

**Figure 4:**
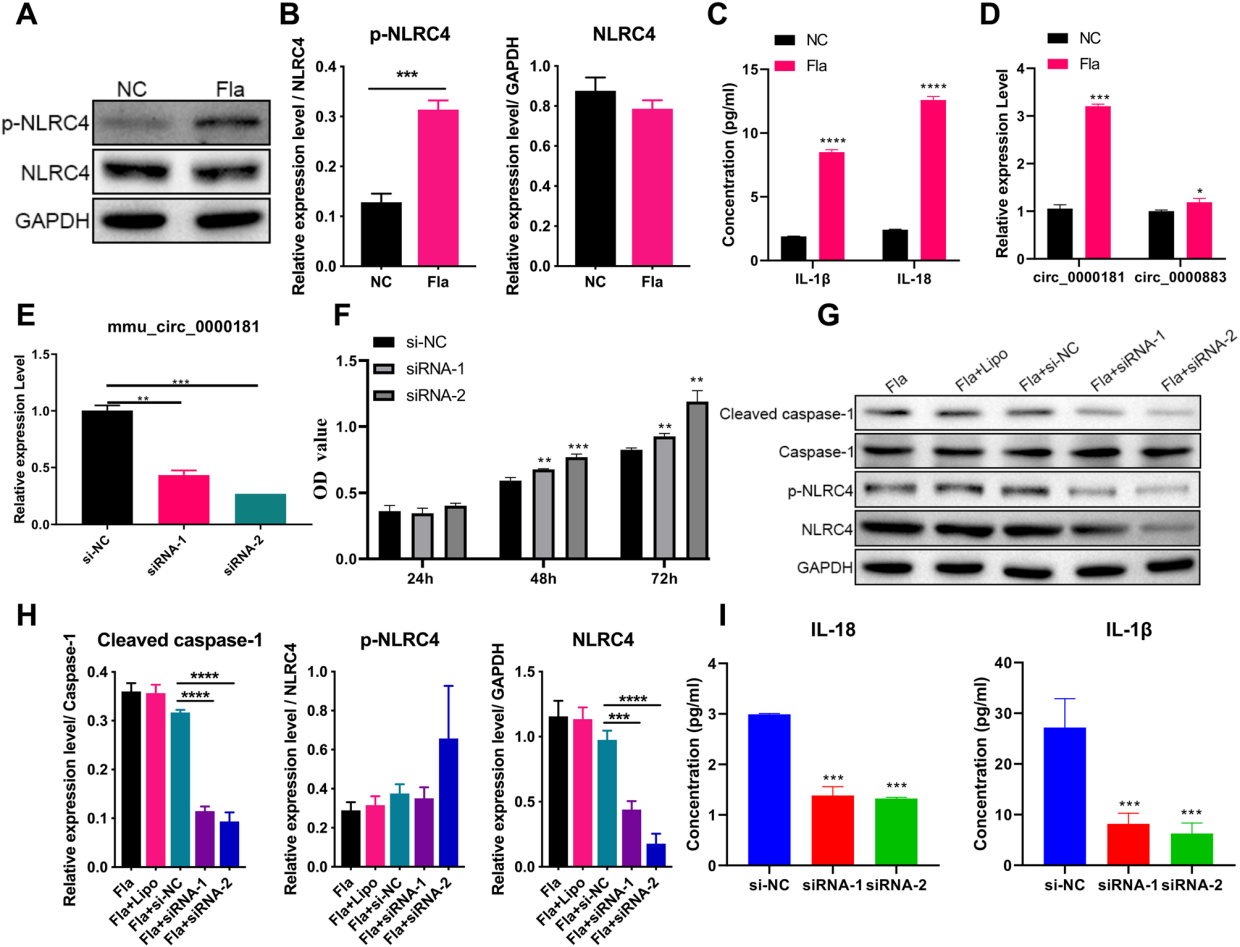
circ_0000181 is involved in NLRC4-induced pyroptosis of MRTEC cells. (**A**) The expression of NLRC4 and p-NLRC4 in MRTEC cells with or without Fla stimulation was detected using WB. The origianl images were cut prior to hybridisation with antibodies during blotting. (**B**) Proteins were quantified based on the gray values of WB results. (**C**) The concentration of IL-1β and IL-18 in MRTEC cells supernatant with and without Fla stimulation (activation for 6 h) was detected using ELISA analysis. (**D**) qPCR analysis was used to measure circ_0000181 and circ_0000883 expression in MRTEC cells with and without Fla stimulation (activation for 6 h). (**E**) qPCR analysis was used to detect circ_0000181 expression under different siRNA interference (Fla activation for 6 h after transfection 48 h). (**F**) Cell proliferation ability was detected using MTS in MRTEC cells with Fla stimulation after transfection with circ_0000181 siRNAs for 48 h. (**G**) The WB analysis was used to detect the expression of NLRC4, p-NLRC4, and Casepase1 in MRTEC cells with Fla stimulation under different siRNA interference. The origianl images were cut prior to hybridisation with antibodies during blotting. (**H**) Quantitative analysis of protein in line with WB data. (**I**) The ELISA analysis was used to determine the secretion of IL-1β and IL-18 in MRTEC cells with Fla stimulation under different siRNA interference. **P < 0.01; ***P < 0.001; ***P < 0.0001. Data are presented as the means ± SD from three independent repetitions.

To explore the interaction mechanism between circ_0000181, circ_0000883, miR-667-5p, and NLRC4, Fla was used to stimulate mouse renal tubular epithelial cells (MRTEC) cells as an in vitro cell model of DN. Then, p-NLRC4, NLRC4 expression, and secretion of inflammatory factors (IL-1β, and IL-18) were detected using WB and Enzyme-linked immunosorbent assay (ELISA). As shown in Fig. 4A–C, the p-NLRC4 expression and inflammatory factors’ secretion was increased and NLRC4 expression did not change in the Fla group compared to the NC group, indicating that our cell model was constructed successfully. Then, circRNAs expression was measured in the Fla and NC groups. As shown in Fig. 4C, circ_0000181 and circ_0000883 expression was higher in MRTEC cells after Fla stimulation than those MRTEC cells without Fla stimulation, in which circ_0000181 expression significantly increased than circ_0000883, so circ_0000181 was chosen for further study (Fig. 4D). To investigate circ_0000181 function, siRNAs target, circ_0000181, was transfected into MRTEC cells for 48 h, then treated with Fla. Results showed that siRNA-1 and siRNA-2 suppressed circ_0000181 expression (Fig. 4E). Additionally, siRNA-1 and siRNA-2 promoted MRTEC cell proliferation at 48 h and 72 h relative to the siRNA negative control (si-NC) group (Fig. 4F), Furthermore, siRNA-1 and siRNA-2 decreased the expression of NLRC4, p-NLRC4, Caspase1, and the secretion of inflammatory factors (IL-1β, and IL-18) than in the si-NC group (Fig. 4G–I). The above results suggested that circ_0000181 inhibition promoted cell viability but reduced cell pyroptosis.

### circ_0000181 regulates the miR-667-5p/NLRC4 Axis in MRTEC cells

**Figure 5:**
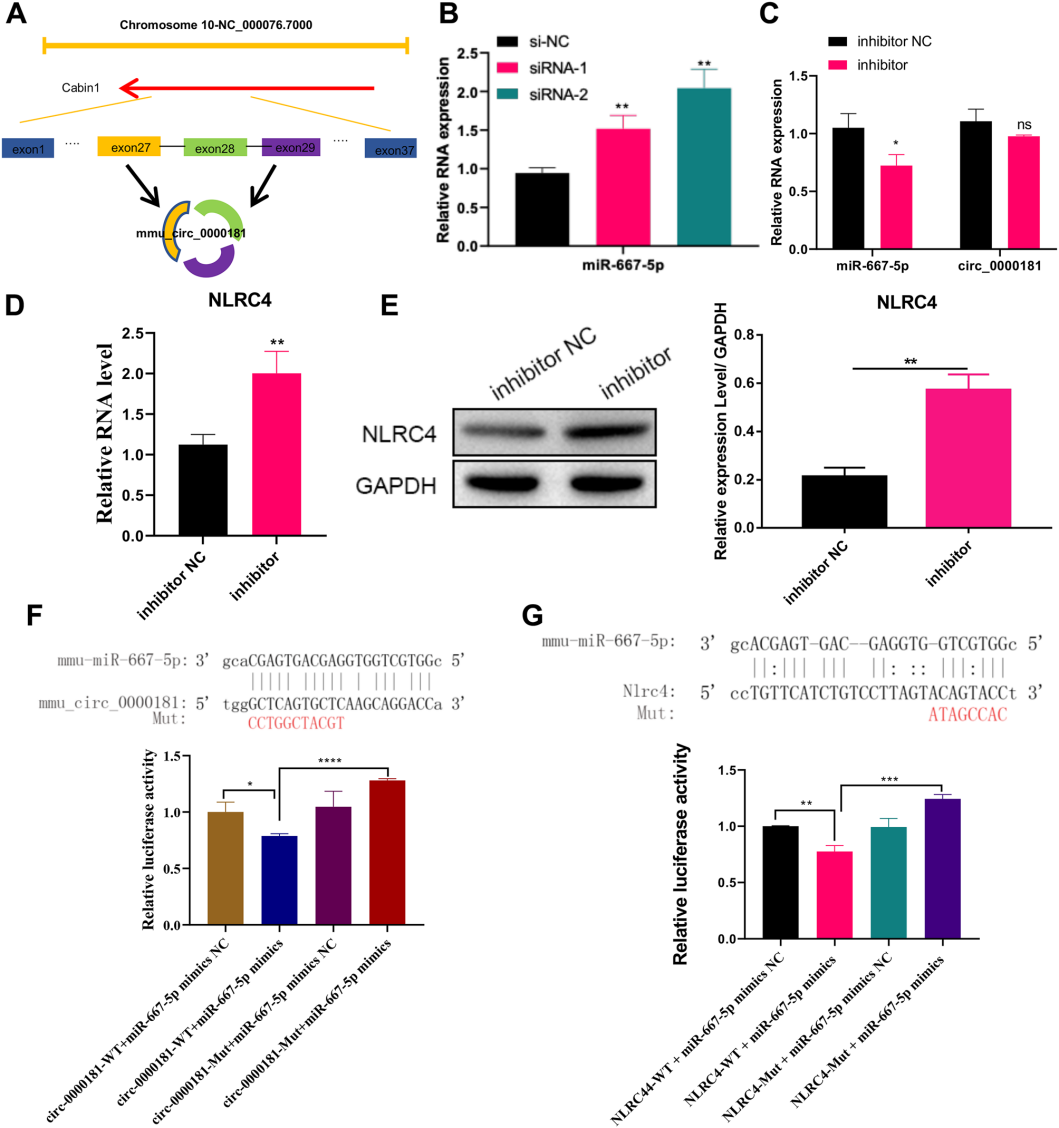
circ_0000181 regulates the miR-667-5p/NLRC4 Axis in MRTEC cells. (**A**) Schematic diagram of shear composition of circ_0000181. (**B**) qPCR analysis was used to measure circ_0000181 and miR-667-5p expression in MRTEC cells under different circ_0000181 interference fragments. (**C**) qPCR analysis was used to evaluate circ_0000181 and miR-667-5p expression in MRTEC cells under miR-667-5p inhibitors interference. (D&E) qPCR (**D**) and WB (**E**) analyses were used to test NLRC4 expression in MRTEC cells under miR-667-5p inhibitor interference. The origianl images were cut prior to hybridisation with antibodies during blotting. (**F**) The prediction binding site of circ_0000181 and miR-667-5p was confirmed using dual-luciferase assay. (**G**) The prediction binding site of miR-667-5p and NLRC4 was confirmed using dual-luciferase assay. *P < 0.05; **P < 0.01; ***P < 0.001; ****P < 0.0001. Data are presented as the means ± SD from three independent repetitions.

According to circbase (http://www.circbase.org/), circ_0000181 was at chromosome 10, and it was composed of the exon of 27, 28, and 29 of the parent gene Cabin 1 (Fig. 5A). To investigate whether circ_0000181 regulated the miR-667-5p/NLRC4 axis, we tested the miR-667-5p expression using qPCR, and the results showed that miR-667-5p expression was significantly increased after circ_0000181 interference, confirming that circ_0000181 regulated miR-667-5p expression (Fig. 5B). Then, we found that miR-667-5p inhibitors did not affect circ_0000181 expression, reduced miR-667-5p expression, and increased its target mRNA NLRC4 expression (Fig. 5C–E). Additionally, we confirmed that circ_0000181 directly binds with miR-667-5p (Fig. 5F), and miR-667-5p directly binds with NLRC4 (Fig. 5G). Based on the results of Figs. 4F and 5, we further verified that circ_0000181 directly binds with miR-667-5p to regulate NLRC4 expression.

### circ_0000181 regulates the pyroptosis process via miR-667-5p/NLRC4 axis in DN

**Figure 6:**
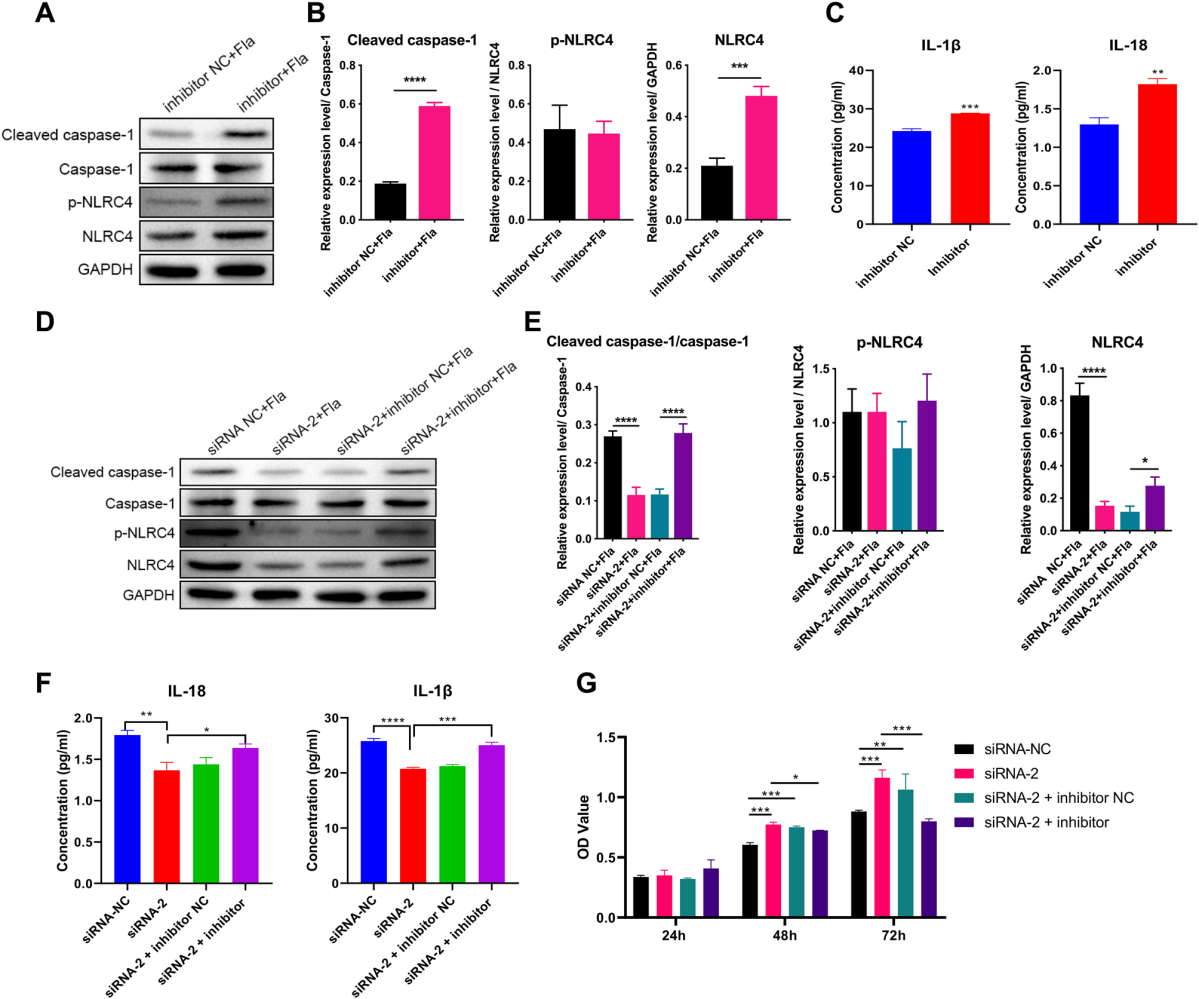
circ_0000181 participates in NLRC4-mediated pyroptosis by regulating miR-667-5p in MRTEC cells. (**A**) The WB analysis was used to evaluate the expression of NLRC4, p-NLRC4, and Caspase1 in MRTEC cells with Fla stimulation under miR-667-5p inhibitor interference. The origianl images were cut prior to hybridisation with antibodies during blotting. (**B**) WB results were further quantitatively analyzed. (**C**) The ELISA analysis was used to determine the secretion of IL-1β and IL-18 in MRTEC cells with Fla stimulation under miR-667-5p inhibitor interference. (**D**) The protein levels of NLRC4, p-NLRC4, and Caspase1 in MRTEC cells with Fla stimulation under siRNA-2 and miR-667-5p inhibitor interference were measured using WB analysis. The origianl images were cut prior to hybridisation with antibodies during blotting. (**E**) Quantitative analysis of WB results. (**F**) ELISA was used to assess the secretion of IL-1β and IL-18 in MRTEC cells with Fla stimulation under siRNA-2 and miR-667-5p inhibitor interference. (**G**) The MTS analysis was used to analyze the MRTEC cells’ proliferation ability with Fla stimulation under siRNA-2 and miR-667-5p inhibitor interference. *P < 0.05; **P < 0.01; ***P < 0.001; ****P < 0.0001. Data are presented as the means ± SD from three independent repetitions.

To clarify miR-667-5p effect on NLRC4-mediated pyroptosis, we transfected the MRTEC cells with miR-667-5p inhibitors, then stimulated them with Fla for six hours. The results showed that miR-667-5p inhibitors promoted NLRC4, p-NLRC4, and Caspase1 expressions (Fig. 6A,B), and IL-1β as well as IL-18 contents in the supernatant were upregulated (Fig. 6C), suggesting that the NLRC4-mediated pyroptosis pathway was activated. Then, the role of circ_0000181 in this process was examined, siRNA-2 was transfected into MRTEC cells alone or in combination with siRNA-2 and miR-667-5p inhibitors. WB results confirmed that siRNA-2 inhibited NLRC4, p-NLRC4, and Caspase1 expressions, while miR-667-5p inhibitors could rescue the downregulation induced by siRNA-2 (Fig. 6D,E). Additionally, miR-667-5p inhibitors improved the decrease in IL-1β and IL-18 expression induced by siRNA-2 (Fig. 6F). Through the MTS cell proliferation assay, it was found that the activity enhancement of MRTEC cells induced by siRNA-2 could be inhibited by miR-667-5p inhibitors (Fig. 6G). Collectively, circ_0000181 regulates the pyrolysis process via miR-667-5p/NLRC4 axis in DN.

## Discussion

DN is a major cause of death of patients with diabetes, and the current treatment of diabetic nephropathy remains a huge challenge. Recently, research on DN pathogenesis has also received extensive attention^21^. Inflammation is widely believed to be involved in DN development, and various studies have confirmed the accumulation of inflammatory cells and pro-inflammatory factors in the kidneys of patients with DN and mouse models^22–24^. The NLRP3 inflammasome is widely recognized as having the potential to target DN^4,25^. Interestingly, our previous studies found that NLRC4 inflammasome expression is abnormally increased in the kidneys of patients with DN^5^. Additionlly, there are few studies on the relationship between NLRC4 inflammasomes and DN development. In this study, STZ induced DN model was established to analyze the activation mechanism of NLRC4^5,26^. Further experimental results showed that NLRC4 was activated in the DN model to promote the release of the inflammatory factors, IL-1β and IL-18. Our results support the presence of NLRC4 activation in DN^5^.

NLRC4 is a pathway independent of NLRP3^27^. After activation, NLRC4 mediates the release of inflammatory factors, such as IL-1β and IL-18, resulting in cell pyrolysis. However, the released verification factors promote inflammatory response and help clear pathogens^28^. However, excessive or low levels of inflammatory factors cause acute or chronic tissue damage^29,30^. Inhibition of NLRC4 activation has a potential therapeutic effect on LPS-induced lung injury^29,30^. Additionlly, inhibiting NLRC4 expression or Caspse1 activation inhibits glial cell death in stroke^31^ and intestinal epithelial injury^32^. Combined with the previous research and the findings of this study, we support that NLRC4 activation played an important role in the occurrence and development of DN injury.

CircRNA regulates gene expression mainly through competitive binding of miRNA, leading to inhibition of miRNA’s inhibitory effect on downstream target mRNA. In DN, only some circRNA functions have been revealed. For example, circRNA_15698, Circular RNA HIPK3, and circ_0000491 have been reported to promote the accumulation of glomerular mesangial cells extracellular matrix and promote DN progress^14,16^. Additionally, circRNA function in DN remains poorly understood. In this study, we observed that NLRC4 was activated in the kidney tissue of DN mice. In terms of mechanism, many mechanisms regulate empty NLRC4 activation, and the circRNA-miRNA pathway may regulate NLRC4. Therefore, to explore the potential circRNA-miRNA pathway upstream of NLRC4, we predicted the target miRNAs of NLRC4, and on this basis, predicted the target circRNAs of these miRNAs to map the competitive inhibition network of circRNA-miRNA-NLRC4. We predicted candidate miRNA targets for circRNA, including circ_0000181, mmu_circ_0000883, mmu_circ_0001055, and mmu_circ_0001477. These four circRNAs were upregulated in the kidney tissue of DN mice, suggesting that they play an important role in DN. Among them, we proved that circ_0000181 and circ_0000883 were real rings and upregulated in DN mice. Presently, the function of these two circRNAs in DN remains unclear. Existing studies have shown that circ_0000181 is downregulated in the plasma of patients with gastric cancer, and correlated positively with the differentiation of gastric cancer cells and carcinoembryonic antigen^14,16^. Additionally, it has been reported that it may be related to the muscle growth and development of cattle breeds^33^. However, their function in DN is unclear. Our cell experiments demonstrated that circ_0000181 promoted NLRC4 inflammasome activation, while interfering with circ_0000181 expression significantly inhibited NLRC4 inflammasome activation. Our research expanded the biological function of circ_0000181 regulating DN to promote renal tubular epithelial cell pyrolysis, and further supported the involvement of circRNAs in regulating DN.

Through bioinformatics analysis, we predicted multiple pathways that circ_0000181 regulates NLRC4, including miR-667-5p, miR-6981-5p, miR-6914-5p, miR-7023-5p, and miR-669e-5p. However, among these miRNAs, only miR-667-5p was downregulated in the tissues of DN mice. Additionally, at the cellular level, functional analysis showed that only miR-667-5p reversed the circ_0000181 effect on NLRC4, indicating that circ_0000181-miR-667- 5p pathway is an important pathway that promotes NLRC4 activation in DN.

Previous studies have shown that miR‐667‐5p is upregulated in the cerebral cortex and hippocampus exposed to PM2.5^34^. Rno-miR-667-5p is downregulated in the muscles of rats with spinal cord injury and is predicted to be involved in the pathogenesis of nerve injury and the reversal effect of neurotrophin-3 on myeloid injury^35^. Additionally, miR-667-5p is highly expressed in the kidney tissue of pregnant mice^36^. Although miR-667-5p has been reported to be differentially expressed in various disease settings, the current miR-667-5p function is unclear. Here, we propose that miR-667-5p is involved in the DN process, and by targeting NLRC4, it inhibits NLRC4 inflammasome activation and inhibits the proliferation of renal tubular epithelial cells.

However, more experiments are needed to confirm these findings in clinical samples. In addition, distinguishing the protein level expression levels of IL-1β and IL-18 between renal tubule interstitium and glomeruli maybe help us to know which one structure of kidney was affected most in DN model, therefore the localization of protein will be conducted in the future study.

## Materials and methods

### Sample collection and DN model construction

The Ethical Committee of Guangzhou Forevergen Medical Laboratory Animal Center (approval no: IACUC-G16054), Guangdong, China, approved all animal experiments. All methods were conducted following relevant guidelines and regulations.

A total of 18 female and male C57BL/6 mice (about 18–22 g) were purchased from Guangdong Experimental Animal Center, and randomly divided into two groups: sham group (N = 6) and DN model (N = 12). The DN model mice were constructed following the previous study^37^ with a few revisions. Briefly, mice were treated with STZ (50 mg/kg, dissolved in sodium citrate, pH 4.5, Sigma-Aldrich, St. Louis, MO, USA) via intraperitoneal injections for five consecutive days. After two weeks, only eight mice with a fasting glucose level above 16 mmol/L were selected for further investigation. The sham group mice were treated with an equal volume of sodium citrate (pH 4.5) via intraperitoneal injections for five consecutive days. At 18 weeks, all mice were killed using the CO_2_ method. Additionally, two kidneys were separated, one was fixed in 4% paraformaldehyde, the other was maintained in liquid nitrogen.

### H&E and PAS staining

For H&E staining, the kidney tissue was dehydrated, embedded in paraffin, and cut into 4-μm thick sections. Sections were then incubated with xylene for 20 min and dewaxed in absolute alcohol, 95% alcohol, 85% alcohol, and 75% alcohol for five minutes each. After washing with distilled water for five minutes, slices were stained with hematoxylin and counterstained with eosin for one minute each, followed by dehydration and sealing. Five 200-fold high-power fields were randomly selected from the same section.

For PAS staining, the kidney tissue was first fixed in 96% alcohol for ten minutes. Then, the samples were incubated in 0.1% periodic acid for ten minutes. The eyelashes were washed in running tap water for ten minutes and immersed in Schiff’s reagent for ten minutes. Subsequently, the samples were washed in tap water for five minutes, counterstained with Mayer’s hematoxylin for three minutes, washed in tap water for five minutes, and followed by dehydration and sealing. Five 200-fold high-power fields were randomly selected from the same section.

### Immunohistochemistry (IHC)

Sections were incubated with 3% H_2_O_2_ for ten minutes at room temperature, placed in an antigen repair solution (Tris–ethylenediaminetetraacetic acid [EDTA], pH 9.0), and microwaved for 30 min. After that, 50-μL blocked serum was added onto sections and left for 30 min at 37 °C. A further 50-μL anti-IL-18 (#ab71495; Abcam, UK); anti-IL-1β (#12242S; CST, USA) was incubated with sections overnight at 4 °C followed by incubation of section with HRP-conjugated secondary antibodies at 37 °C for one hour. Finally, the DAB solution was used to reveal the immunohistochemical staining and five 200-fold high-power fields were randomly selected from the same section. Image J 1.8.0 was used for quantitative analysis of the IHC signal. No primary antibodies incubation was used as the negative control for IHC.

### Next-generation sequencing

Differentially expressed circRNAs, miRNAs, and mRNAs profiles were characterized using large-scale next-generation deep sequencing methods as described earlier with minor alterations^38^ with the mixed mRNA samples from the kidney tissue of the DN model (n = 4) and sham mice (n = 4). Briefly, total RNAs were extracted using the iPrep™ Trizol™ Plus RNA Kit (#IS10007; Thermo Fishier Scientific, USA) following the manufacturer’s instructions. Following RNA quality validation via agarose gel electrophoresis and OD260/280 value, the cDNA library was established with 0.2-μg RNA using the SmartPCR cDNA kit (#634925; CLONTECH Laboratories, Japan) following the manufacturer’s instructions. After removing the adaptor using RsaI digestion (Thermo Fisher Scientific, USA), cDNA samples were subjected to sonification fragmentation, profiling with Agilent Bioanalyzer, Illumina library establishment, and quality verification using an Agilent Bioanalyzer 2100 machine, and finally sequenced using the Illumina HiSeq2000 system. Then, relative RNA expressional levels were determined using the Cufflinks 2.0.2 software. Significantly different expressed genes were defined by a Log2 (fold change) ≥ 0.67 or log2 (fold change) ≤  − 0.67, a P-value of < 0.01. KEGG analysis was conducted to investigate the functions of differentially expressed mRNAs using the Database for Annotation, Visualization and Integrated Discovery.

### CircRNA identification

Agarose gel electrophoresis and Sanger sequencing were required to verify the circular structures of mmu_circ_0000181 and mmu_circ_0000883. According to the sequence information from circbase (http://www.circbase.org/), the divergent and convergent primers were designed. The primer sequences are listed in Table 1. cDNA (complementary DNA) and gDNA (genomic DNA) were extracted from the kidney based on the manufacturer’s protocol. Then, cDNA and gDNA were, respectively, for PCR amplification. Also, the amplified products were further examined via agarose gel electrophoresis and Sanger sequencing.

**Table 1:** The primer sequence in this study.

Primer name	Sequence (5'–3')
mmu_circ_0000181-CF1	CTACAGCAAGACTCACCGGA
mmu_circ_0000181-CR1	TCCTGGCTTCGGTGTTGAA
mmu_circ_0000181-LF	CCTCTGCTTACATCCCCAGC
mmu_circ_0000181-LR	TCAAGCACAGGCAGAAGGAG
mmu_circ_0000883-CF1	AGACCCCTCCAAGCTCTGTA
mmu_circ_0000883-CR1	ATGATGGGGTGTGAGAGTCG
mmu_circ_0000883-LF	GCCTCCCCGTTACCAAAAGA
mmu_circ_0000883-LR	AGGATGGTGACCCTTTGCTG
mmu_circ_0001055-CF1	CACCAGGCAGCCTCATTG
mmu_circ_0001055-CR1	AGAGTTCGGCAATGGGGTTA
mmu_circ_0001055-LF	GTGGTGGCCAAGATTCAGGA
mmu_circ_0001055-LR	TGGGAGGGTGAGGAAGTCAG
mmu_circ_0001477-CF1	AGTCTGGCTGCTAAATTGGC
mmu_circ_0001477-CR1	CCTGAATATTGGCAACCGGG
mmu_circ_0001477-LF	GCAGAAGTGAAGGGGACGAT
mmu_circ_0001477-LR	CGAGAACTCAGGCTGCTGTA
M-GAPDH-LF	AGGTCGGTGTGAACGGATTTG
M-GAPDH-LR	TGTAGACCATGTAGTTGAGGTCA
M-GAPDH-CF	TGACAATGAATACGGCTACAGC
M-GAPDH-CR	CACACCGACCTTCACCATTTAC
mmu-miR-667-5p-F	AACAATCGGTGCTGGTGGA
mmu-miR-667-5p-RT	GTCGTATCCAGTGCAGGGTCCGAGGTATTCGCACTGGATACGACCGTGCT
mmu-miR-6914-5p-F	AACAAGTCCTGGGGTGGTG
mmu-miR-6914-5p-RT	GTCGTATCCAGTGCAGGGTCCGAGGTATTCGCACTGGATACGACTCTGTG
mmu-miR-6981-5p-F	AACAAGGTGAGGAGAAGGAAGAG
mmu-miR-6981-5p-RT	GTCGTATCCAGTGCAGGGTCCGAGGTATTCGCACTGGATACGACGCCTTC
mmu-miR-669e-5p-F	AACACGCTGTCTTGTGTGTG
mmu-miR-669e-5p-RT	GTCGTATCCAGTGCAGGGTCCGAGGTATTCGCACTGGATACGACATGAAC
mmu-miR-7023-5p-F	AACAAGATGGGGGAGCTGG
mmu-miR-7023-5p-RT	GTCGTATCCAGTGCAGGGTCCGAGGTATTCGCACTGGATACGACACGCCC
Universe R	GTGCAGGGTCCGAGGT
M-NLRC4-F	AATTCAGATGGGCAGACAGG
M-NLRC4-R	GAGCCCTATTGTCACCAGGA
M-caspase-1-F	GGCACATTTCCAGGACTGACTG
M-caspase-1-R	GCAAGACGTGTACGAGTGGTTG
U6-F	CTCGCTTCGGCAGCACA
U6-R	AACGCTTCACGAATTTGCGT

### Construction of circRNAs–miRNAs–NLRC4 network

Based on the circRNA ID in circBase for the ceRNA targeting NLRC4 gene (Table S4), the length of circRNA is between 350 and 3000 bp, and the number of circRNA and miRNA binding sites is greater than 1, and the circRNA is screened to make circRNAs–miRNAs–NLRC4 network. Then the circRNAs–miRNAs–NLRC4 network was visualized using cytoscape software.

### Cell culture and flagellin treatment

The MRTEC were obtained from cellcook (Guangzhou, China). Cells were cultured at 37℃ in DMEM (#41500034; GIBCO, USA) containing 10% fetal bovine serum in a humidified cell culture cabinet supplied with 5% CO_2_.

Flagellin (Fla, SRP8029-10UG, Sigma) was used to treat MRTEC cells to accomplish the inner environment of DN. The experiment protocol was conducted following the previous study with a few revisions^39,40^. MRTEC cells were cultured and transferred to a 96-well plate. Also, 15-μL DOTAP transfection reagent was added into 35-ul serum-free medium for mixing. Then, 50-μL Fla was added to a 50-μL serum-free medium for mixing. The two were mixed and incubated at room temperature for 20 min before adding 850-μL medium. When the cell density was 70%, the medium was discarded, 900-μL solution was added to a six-well plate, of which 100 μL was added to a 96-well plate, and samples were collected after treatment for 6 h.

### Quantitative PCR (qPCR)

The relative RNA levels were assessed using the quantitative PCR (qPCR). Briefly, the total RNA samples were isolated using the TRIzol Reagent (#R0016; Beyotime, Beijing, China) following the manufacturer’s instructions. Approximately, 3.0-μg RNA sample per cell group was subjected to the cDNA library synthesis, using the Bestar qPCR RT kit (#2220; DBI Bioscience, Germany) following the manufacturer’s instructions. Gene expressional levels were finally determined using the real-time quantitative PCR method with the Bestar qPCR Master Mix kit (#2043; DBI Bioscience, Germany) following the manufacturer’s instructions. The qPCR was conducted via predenaturation for two minutes at 94 °C, followed by 41 cycles of 94 °C for 20 s, 58 °C for 20 s, and 72 °C for 20 s. Then, relative RNA levels were measured using the 2^–ΔΔCt^ method. GAPDH served as the internal standard of circRNAs and mRNAs. U6 was the internal standard of miRNAs. The sequences of the primers used for qPCR are outlined in Table 1.

### Western blotting (WB)

Total protein samples were extracted using the Tissue or Cell Total Protein Extraction Kit (#C510003; Sangon Biotech, China) following the manufacturer’s instructions. The protein concentration was determined using the Modified Bradford reagent (#C100530; Sangon Biotech, China). Approximately 30-μg proteins per cell group were then boiled at 100 °C for five minutes, separated with 10% SDS-PAGE, transferred onto 0.45-μm PVDF membrane (Millipore, USA), blocked with 5% lipid-free milk solution, incubated with primary antibodies overnight at 4 °C, incubated with horseradish peroxide reductase-conjugated secondary antibodies, and finally developed with the enhanced chemiluminescence (ECL) substrates (Thermo Fisher Scientific, USA). Glyceraldehyde-3-phosphate dehydrogenase (GAPDH) served as the internal standard. The primary antibodies used in this study include anti-NLRC4 (#PA5-88997; Invitrogen, USA), anti-PNLRC4 (#MA5-31846; Invitrogen, USA), anti-Caspase1 (#22915-1-AP; Proteintech, USA), anti-Caspase1 p20 (#AG-20B-0042-C100; Adipogen, USA), and anti-GAPDH (#60004-1-lg; Proteintech, USA).

### MTS analysis

Cell proliferation of MRTEC cells was evaluated by the MTS method using the MTS Assay Kit (Colorimetric, #ab197010; Abcam, UK) following the manufacturer’s instructions. Briefly, the treated MRTEC cells with a density of 4 × 10^4^ cells/ml were seeded into a 96-well plate. After 24, 48, 72 h, the cells were incubated with MTS reagent for four hours at 37℃, and OD values were measured at an absorbance of 490-nm.

### ELISA

The concentration of inflammatory factors in the cell supernatant was analyzed using ELISA kits (CUSABIO, China) targeting IL-18 (#CSB-E04609m) and IL-1β (#CSB-E08054) following the manufacturer’s instructions.

### circRNA and miRNA inhibitors and cell transfection

The circ_0000181-siRNA-1 (sense: 5′-CCAAUUUCUUCAACACCGAA-3′, antisense: 5′-UUCGGUGUUGAAGAAAUUGG-3′), circ_0000181-siRNA-2 (sense: 5′-CAAUUUCUUCAACACCGAAGC-3′; antisense: 5′-GCUUCGGUGUUGAAGAAAUUG-3′) and siRNA-NC: (sense: 5′-UUCUCCGAACGUGUCACGUTT-3′, antisense: 5′-ACGUGACACGUUCGGAGAATT-3′) were synthesized by Genepharma (Shanghai, China). For overexpression, the circ_0000181 was constructed by Genepharma in the LV003 vector. Then, siRNAs and recombinant LV003 vectors were introduced into MRTEC cells using the Lipofectamine™ 3000 Transfection Reagent (Invitrogen™) following the manufacturer’s instructions. circ_0000181 expression was detected using qPCR after 48 h transfection.

### Dual-luciferase assay

Wild-type (WT)/mutant-type (Mut) circ_0000181 and NLRC4 were synthesized by Sangon Biotech (Shanghai, China) and cloned into Promega Dual-Luciferase^®^ Reporter (Promega, Beijing, China). Then, MRTE cells were seeded on a 96-well plate at a density of 8000 cells per well, divided into four groups, each with three biological repeats. The following day, WT/Mut circ_0000181 and NLRC4 were transfected with miR-667-5p or NC mimics (synthesized by Genepharma), respectively. The Dual-Glo Luciferase Assay System (Promega) was used to determine the luciferase activity following the manufacturer’s instructions. Fluorescence values were read using a multilabel microplate reader. Finally, the luciferase activity of each group was calculated as: firefly fluorescence value/Renilla fluorescence value.

### Statistical analysis

This study’s quantitative data based on at least three biological repeats were presented as mean ± SD (standard deviation). The differences were evaluated using the Student T-test (two groups) and analysis of variance methods as appropriate. Differences were considered significant when the *P-value was < 0.05; **P-value was < 0.01; ***P-value was < 0.001.

### Ethical statement

All animal experiment were approved by the Ethical Committee of Guangzhou Forevergen Medical Laboratory Animal Center (approval no: IACUC-G16054), Guangdong, China.

### ARRIVE guidelines

The study was conducted in compliance with the ARRIVE guidelines and all methods were conducted in accordance with relevant guidelines and regulations.

## Conclusion

In summary, we observed NLRC4 inflammasome activation and mediated activation of the pyroptosis pathway in the DN mice. Further experiments demonstrated that circ_0000181 promoted NLRC4 expression by silencing miR-667-5p, leading to Caspase1 enzyme activity activation, releasing IL-18 and IL-1β, and aggravating DN progression. Our results revealed the NLRC4 inflammasome in DN from the level of circRNA-miRNA. The mechanism of activation may provide some insights into the formation and treatment of DN.

## Supplementary Information

Supplementary Figures.

Supplementary Figure S1.

Supplementary Table S1.

Supplementary Table S2.

Supplementary Table S3.

Supplementary Table S4.

Supplementary Table S5.

## Data Availability

The raw sequencing data employed in this article has been submitted to the NCBI trace and short-read archive (https://www.ncbi.nlm.nih.gov/sra); BioProject: PRJNA856794. The datasets generated during and/or analyzed during the current study are available from the corresponding author on reasonable request.
