# Supplementary material for: Circ_0000181 regulates miR-667-5p/NLRC4 axis to promote pyroptosis progression in diabetic nephropathy
Source: Sci Rep. 2022 Jul 14;12:11994. doi: 10.1038/s41598-022-15607-7 (PMC9283475; doi:10.1038/s41598-022-15607-7)
Supplement: Supplementary file 1 — Supplementary Figures. [file 41598_2022_15607_MOESM1_ESM.pdf]

Circ_0000181 regulates miR-667-5p/NLRC4 axis to promote pyroptosis progression in diabetic nephropathy

Running title: Circ_0000181 in diabetic nephropathy

Yining Li[1], Weihong Yu[2], Hao Xiong[2], Fang Yuan[2,#]

[1]Center of organ transplantation, the Second Xiangya Hospital of Central South University, Changsha 410011, China
[2]Department of Nephrology, the Second Xiangya Hospital of Central South University, Changsha 410011, China

#Corresponding author:
Fang Yuan
Department of Nephrology, the Second Xiangya Hospital of Central South University, No 139 Renmin Road, Changsha 410011, China
Tel: +86-0731-85292057
Email: yuanfang@csu.edu.cn

Figure 1D

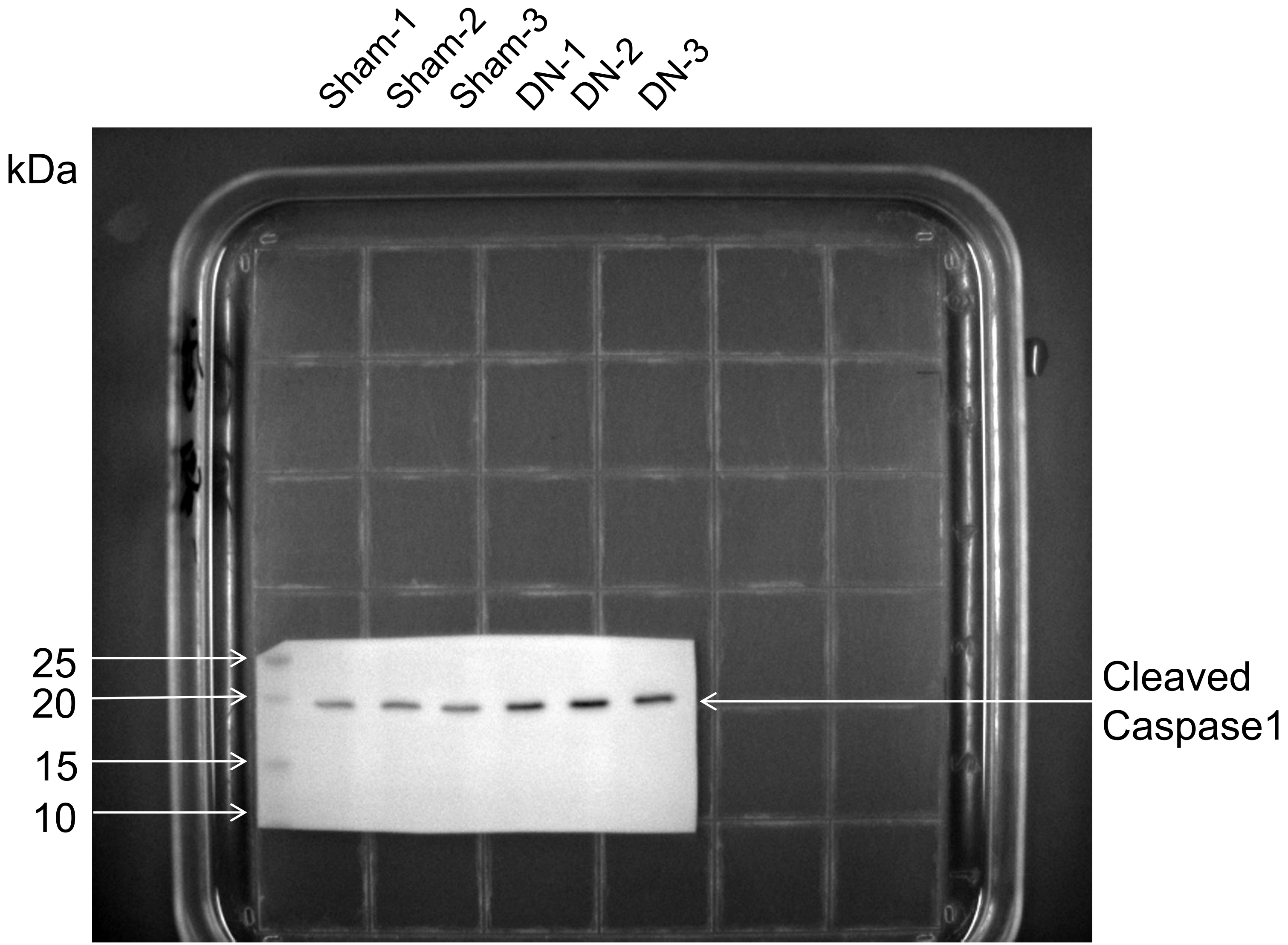

Cleaved Caspase1, AdipoGen, AG-20B-0042-C100, 1:1000, 20~50kD;
anti-Mouse IgG, Jackson, 115-035-003, 1:2000

Figure 1D

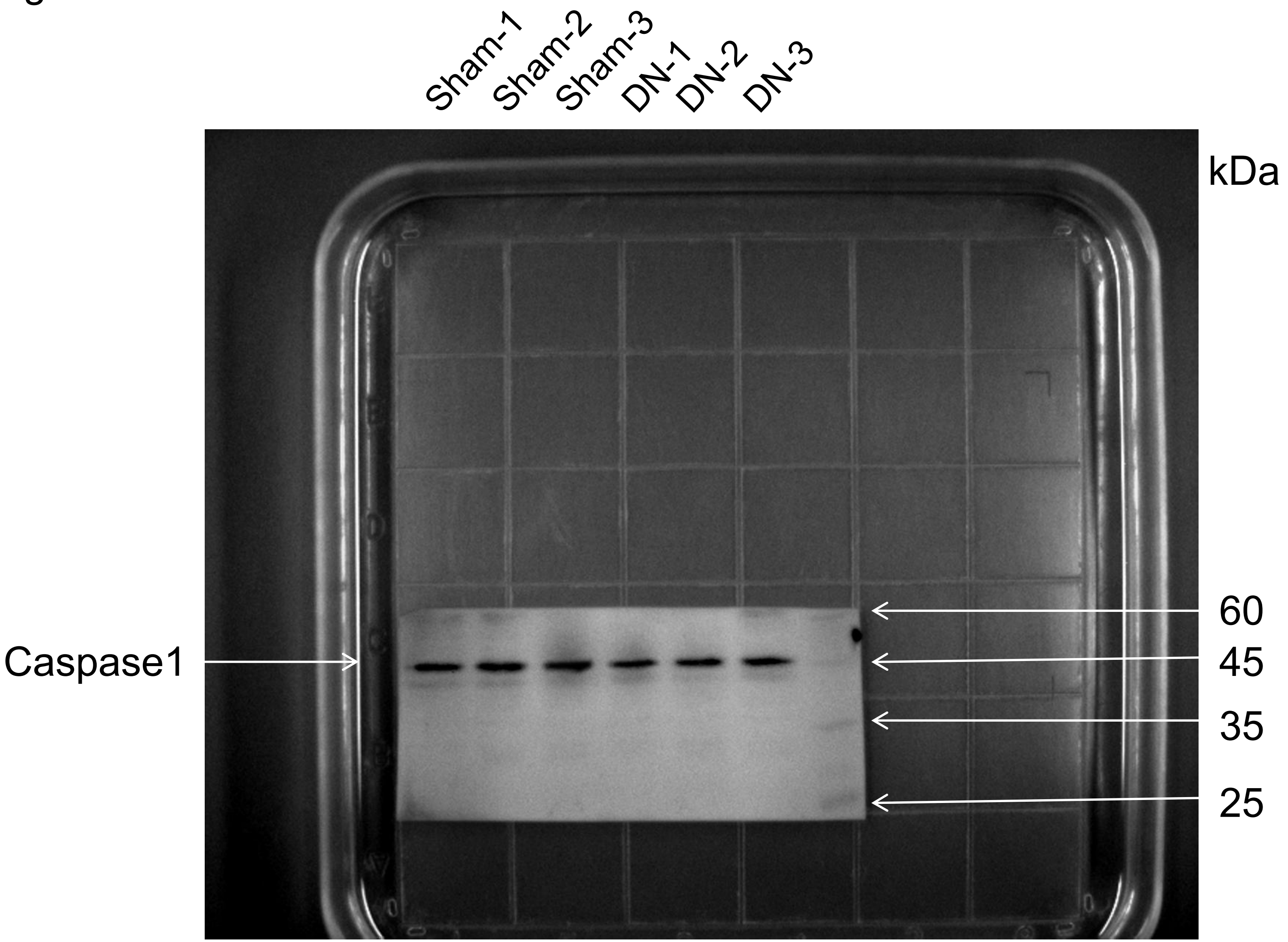

Caspase1, Proteintech, 22915-1-AP, 1:1000, 30/35/45~47kD;
anti-Rabbit IgG, Jackson, 111-035-003, 1:2000

Figure 1D

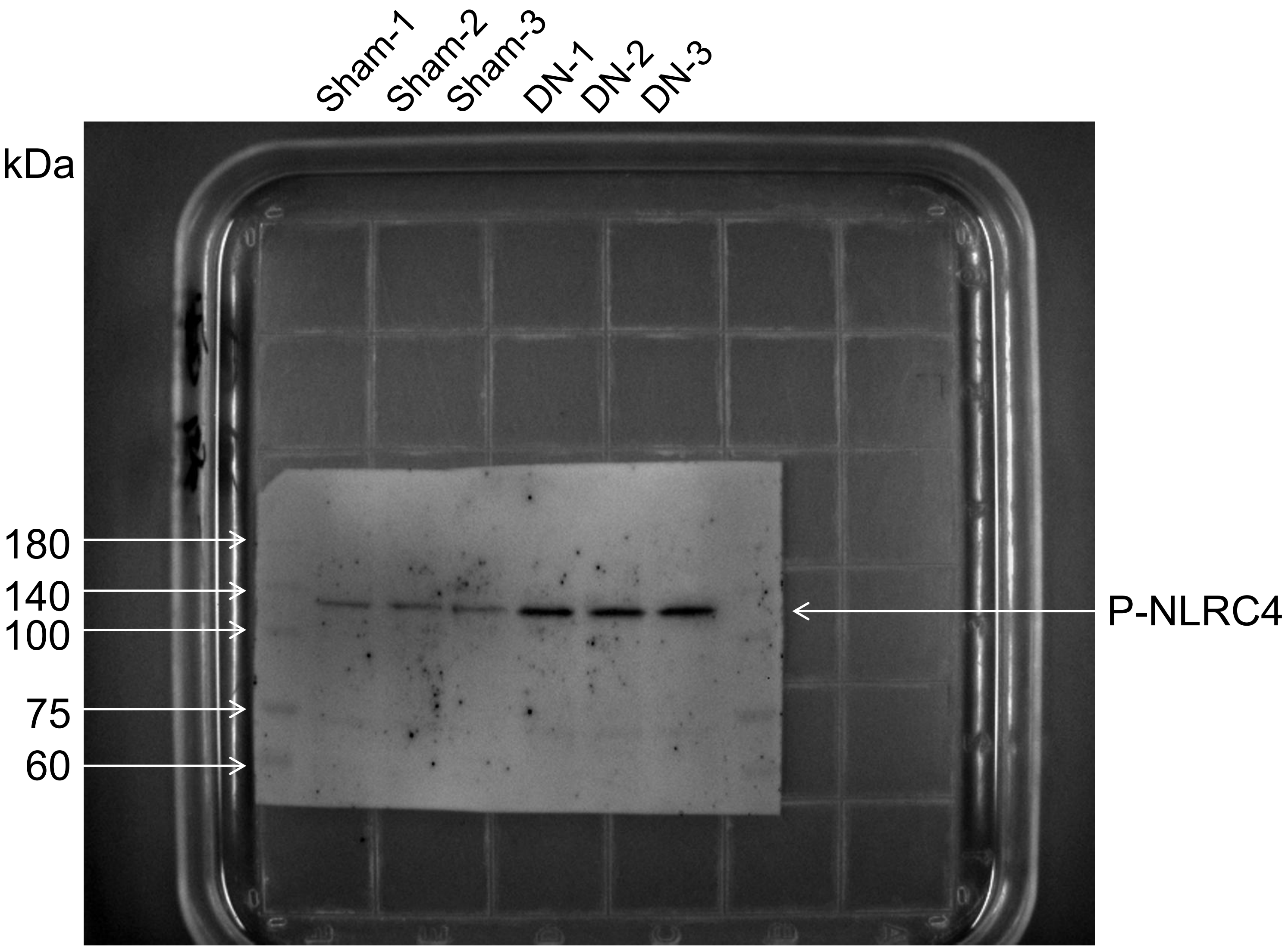

P-NLRC4, Invitrogen, MA5-31846, 1:1000;
anti-Mouse IgG, Jackson, 115-035-003, 1:2000

Figure 1D

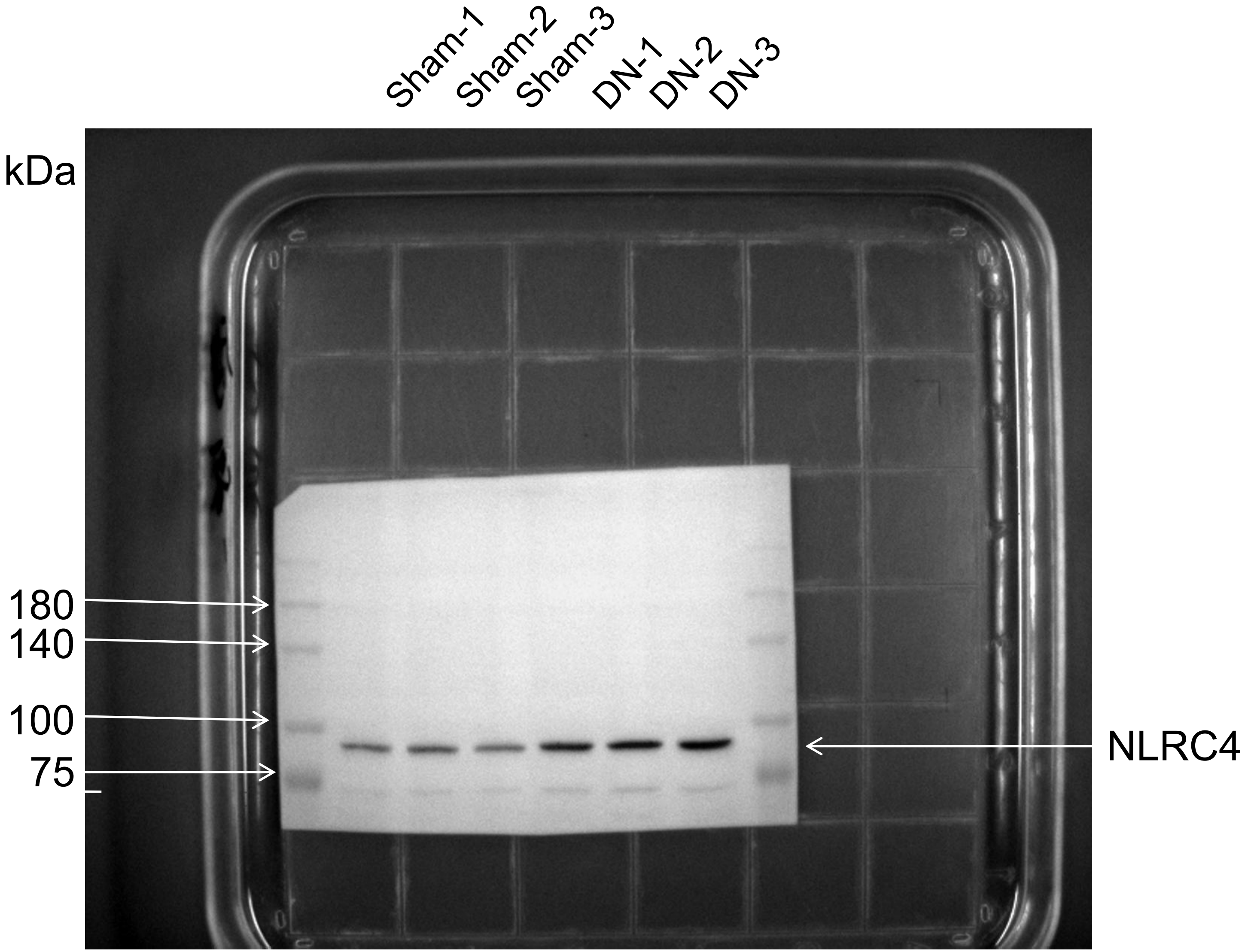

NLRC4, Invitrogen, PA5-88997, 1:1000;
anti-Rabbit IgG, Jackson, 111-035-003, 1:2000

Figure 1D

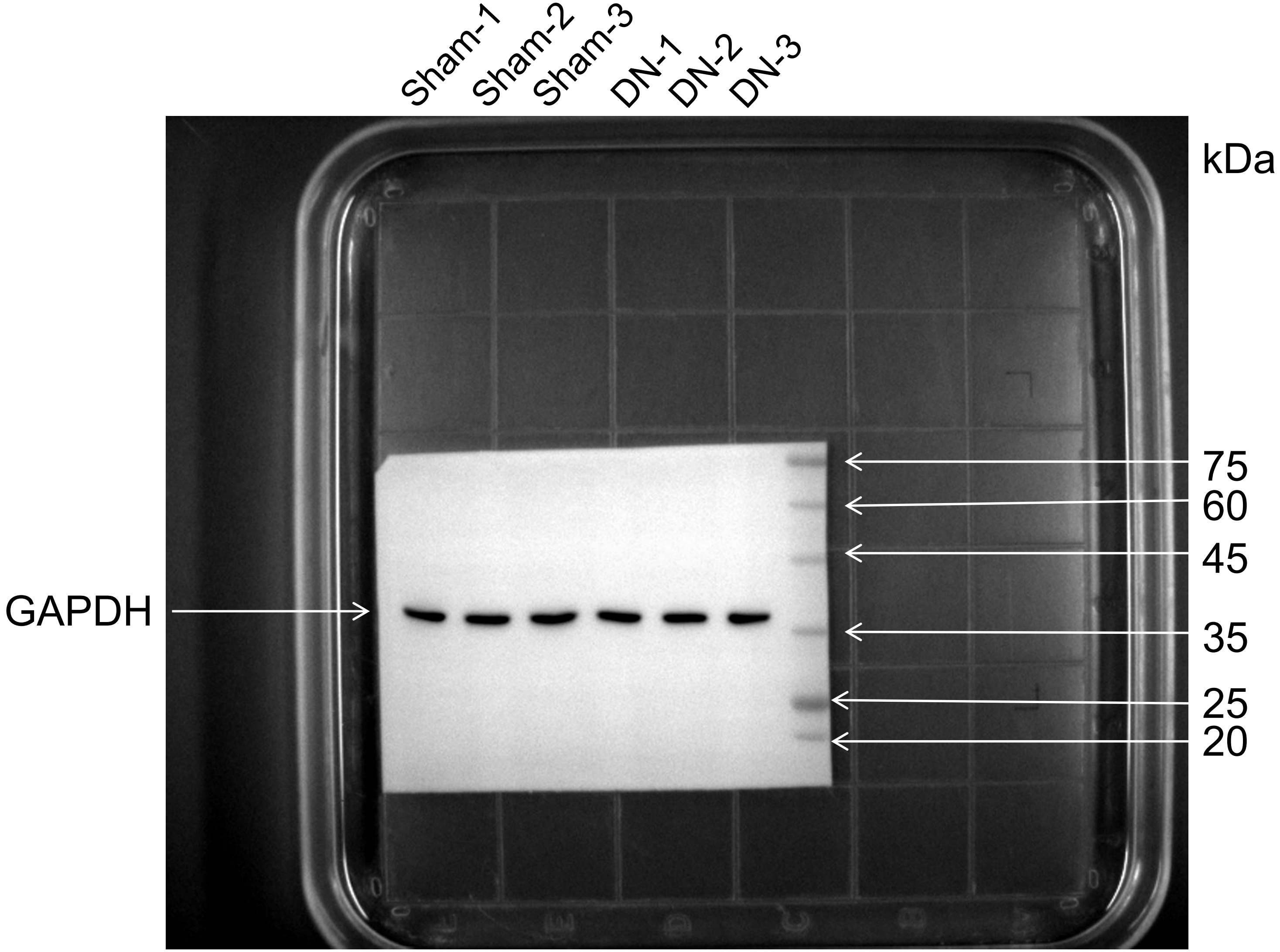

GAPDH, Proteintech, 60004-1-Ig, 1:10000;
anti-Mouse IgG, Jackson, 115-035-003, 1:5000

Figure 3C

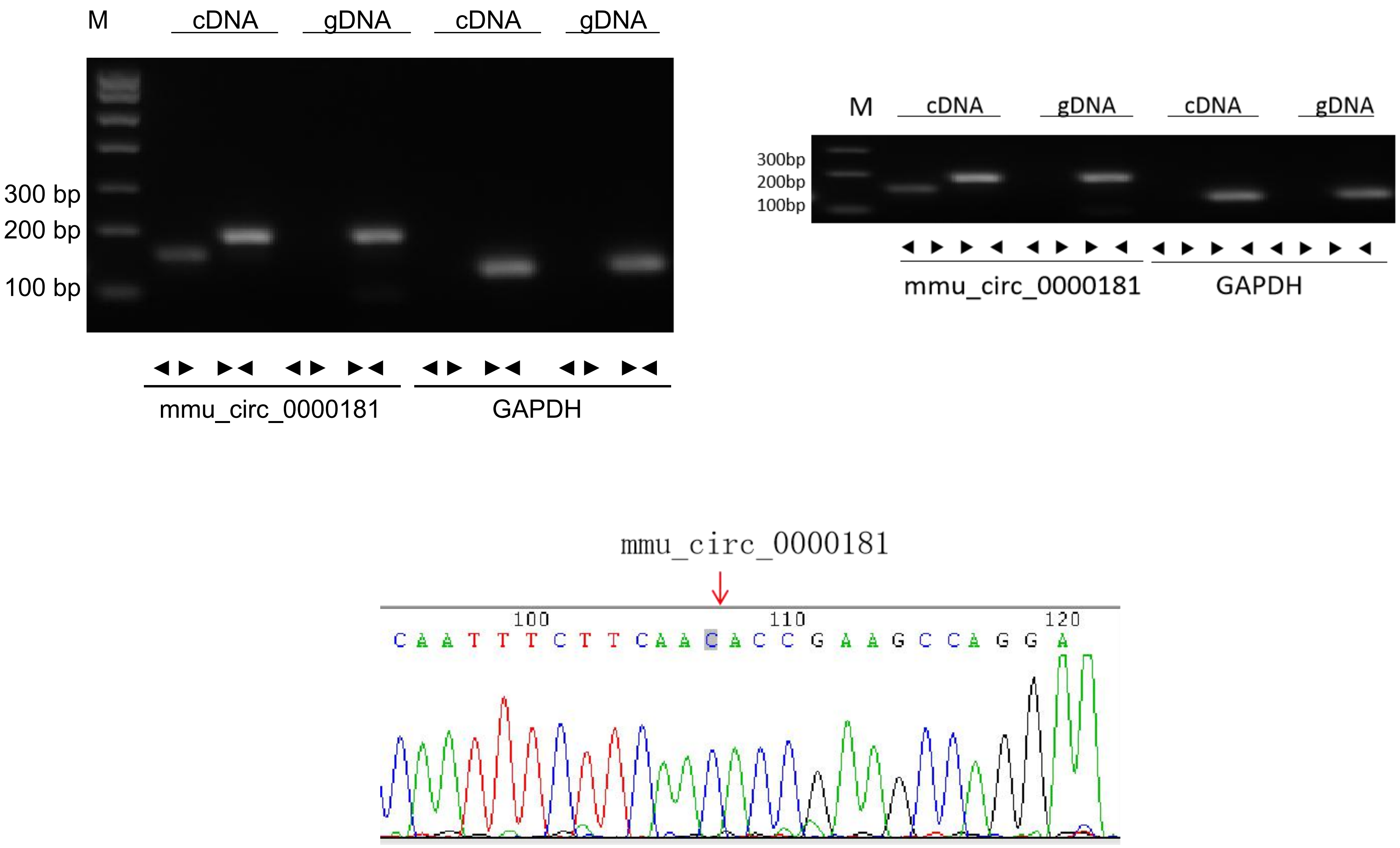

M
cDNA
gDNA
cDNA
gDNA
300 bp
200 bp
100 bp
mmu_circ_0000181
GAPDH
300bp
200bp
100bp
mmu_circ_0000181
100
110
120
C A A T T T C T T C A A C A C C G A A G C C A G G A

Figure 3C

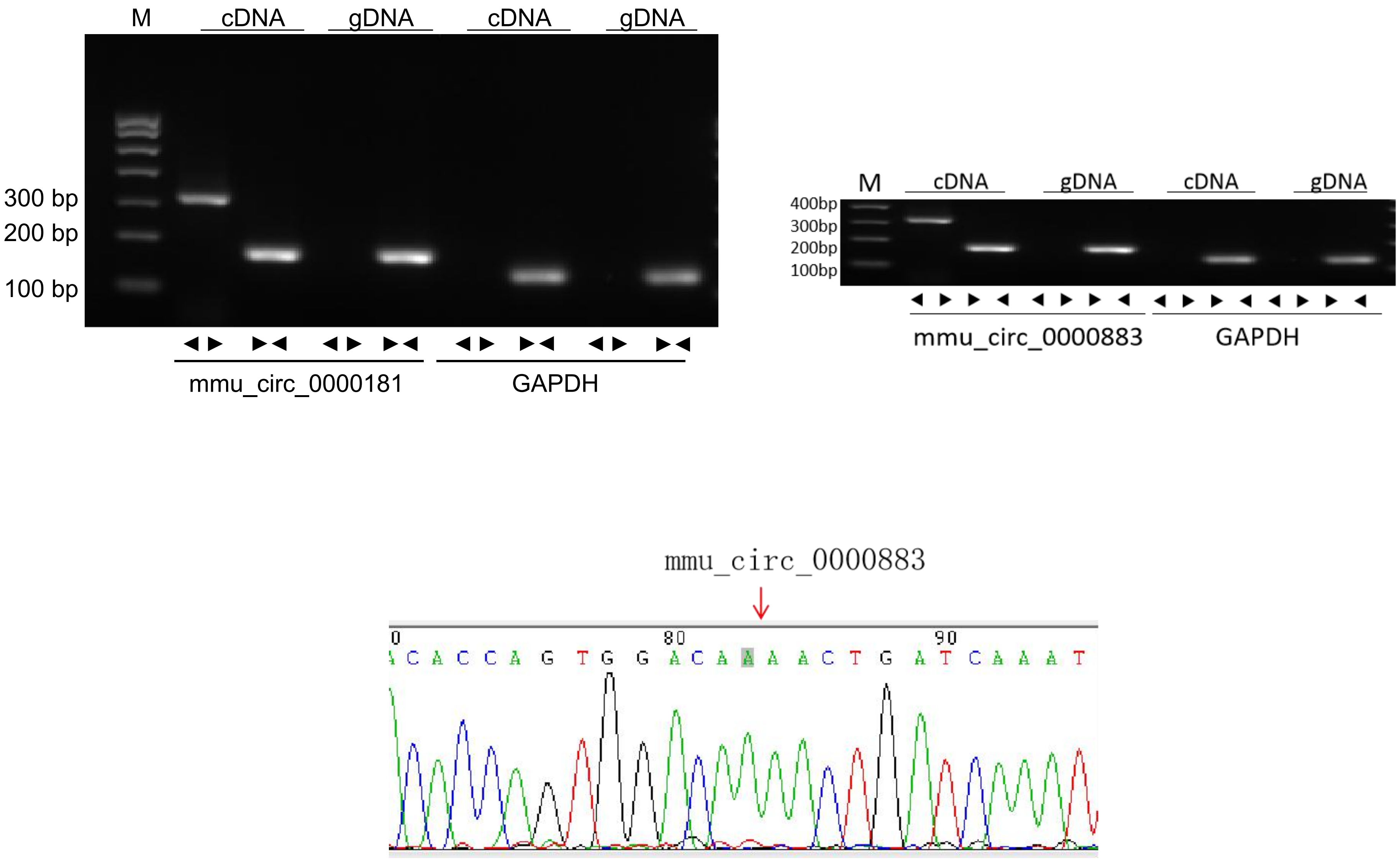
M
cDNA
gDNA
cDNA
gDNA
300 bp
200 bp
100 bp
mmu_circ_0000181
GAPDH
M
cDNA
gDNA
cDNA
gDNA
400bp
300bp
200bp
100bp
mmu_circ_0000883
GAPDH
mmu_circ_0000883
80
90
C A C C A G T G G A C A A A A C T G A T C A A A T

Figure 4A

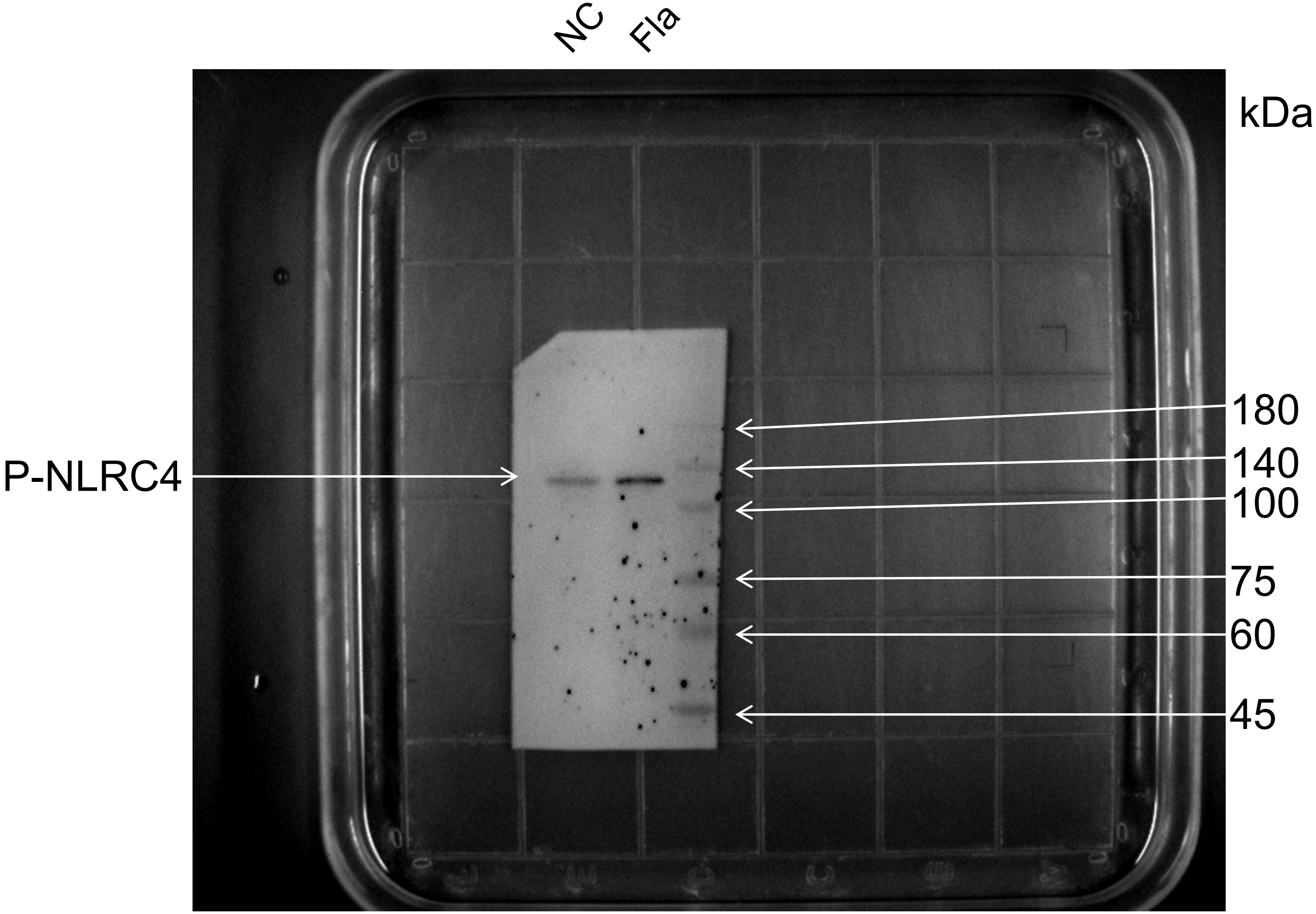

P-NLRC4, Invitrogen, MA5-31846, 1:1000;
anti-Mouse IgG, Jackson, 115-035-003, 1:2000

Figure 4A

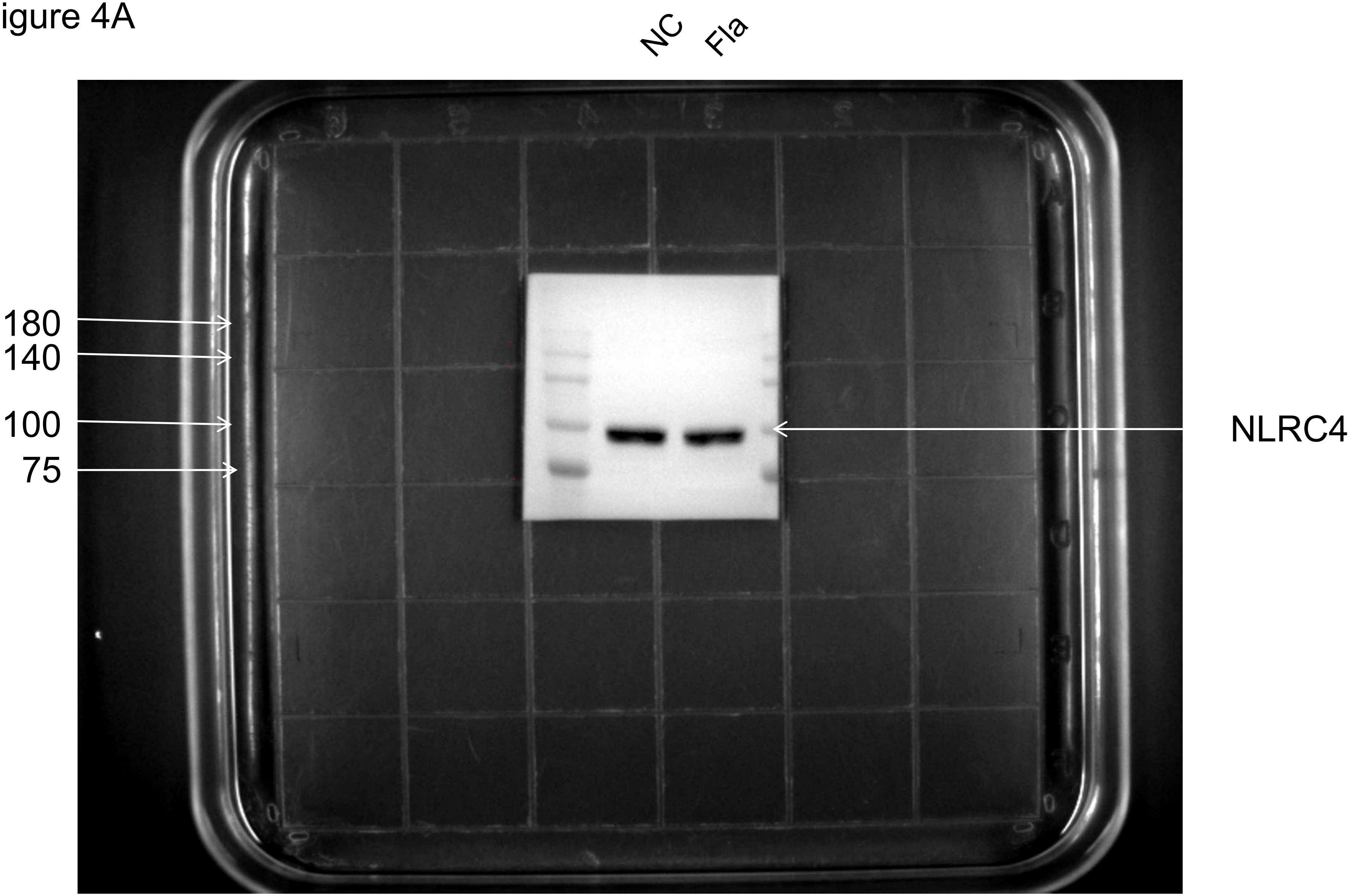

NLRC4, Invitrogen, PA5-88997, 1:1000;
anti-Rabbit IgG, Jackson, 111-035-003, 1:2000

Figure 4A

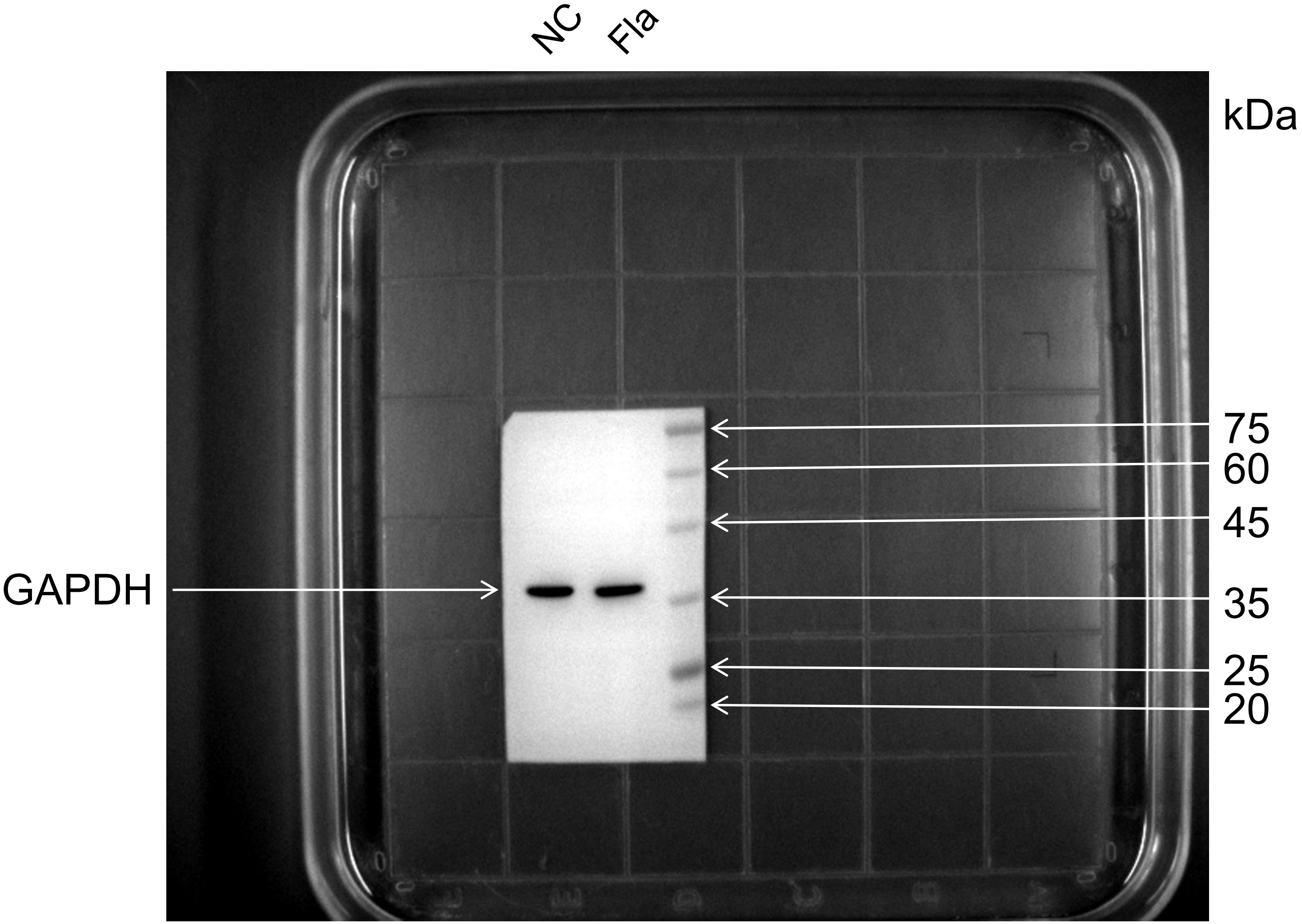

GAPDH, Proteintech, 60004-1-Ig, 1:10000;
anti-Mouse IgG, Jackson, 115-035-003, 1:5000

Figure 4G

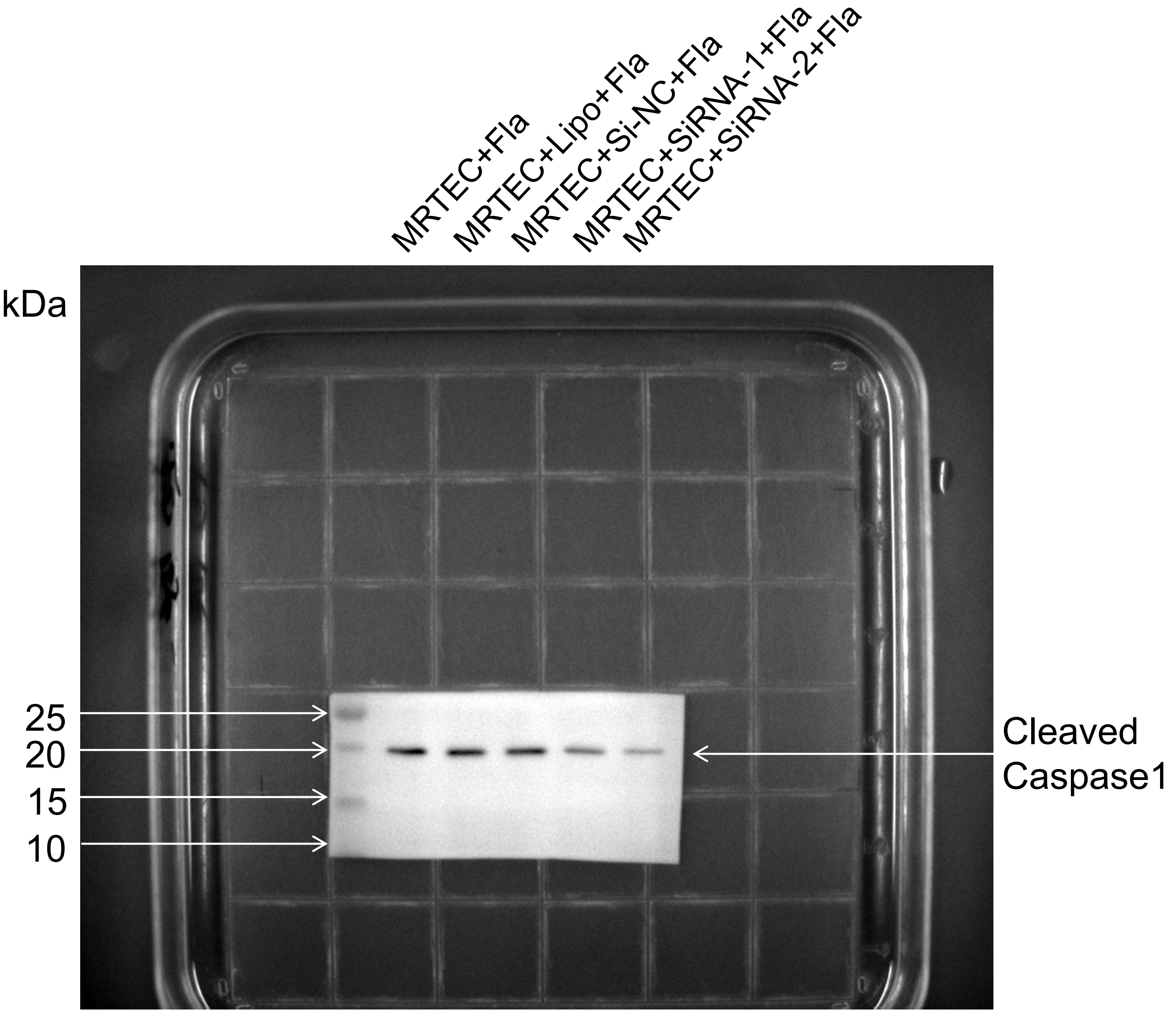

Cleaved Caspase1, AdipoGen, AG-20B-0042-C100, 1:1000, 20~50kD;
anti-Mouse IgG, Jackson, 115-035-003, 1:2000

Figure 4G

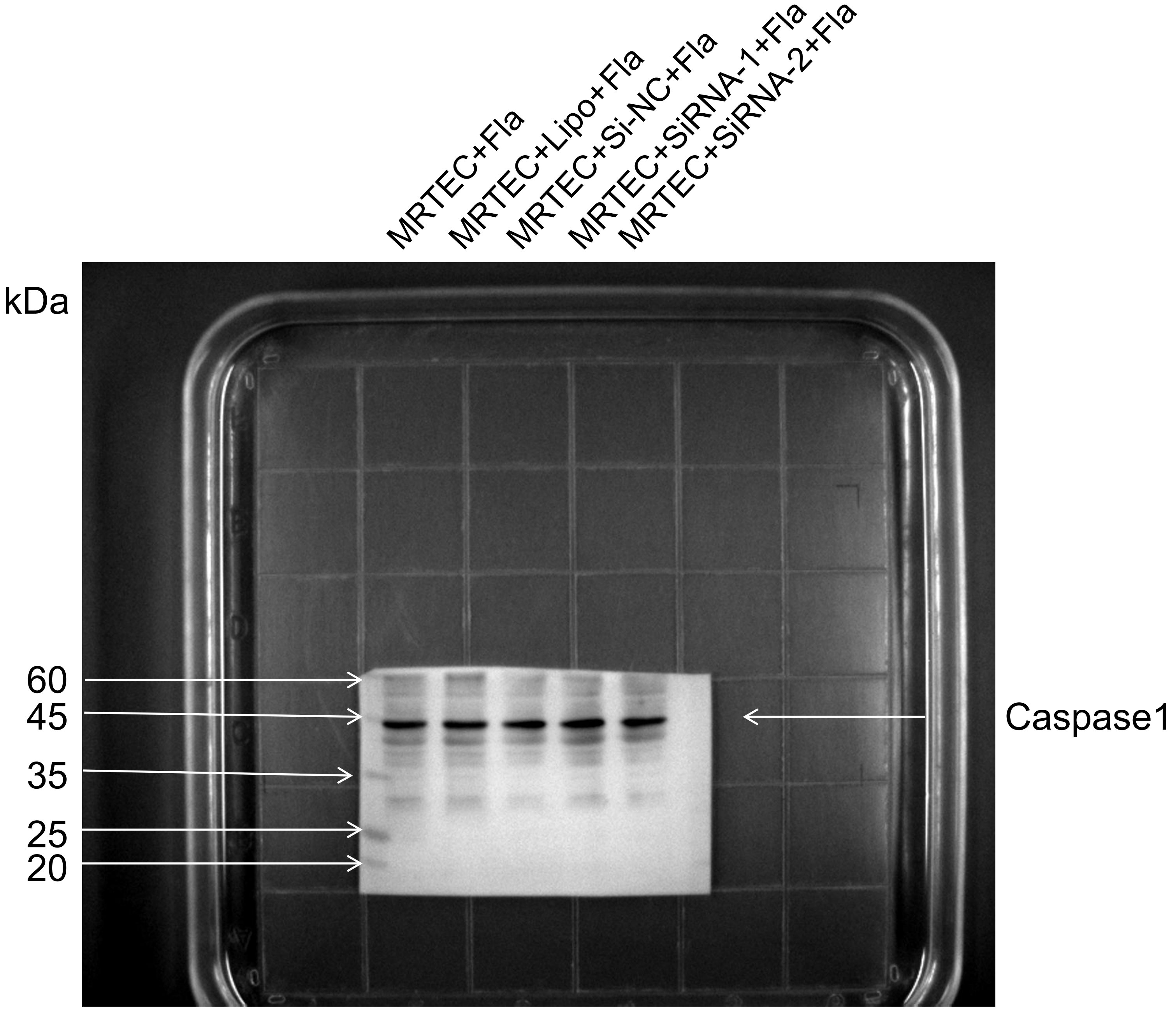

Caspase1, Proteintech, 22915-1-AP, 1:1000, 30/35/45~47kD;
anti-Rabbit IgG, Jackson, 111-035-003, 1:2000

Figure 4G

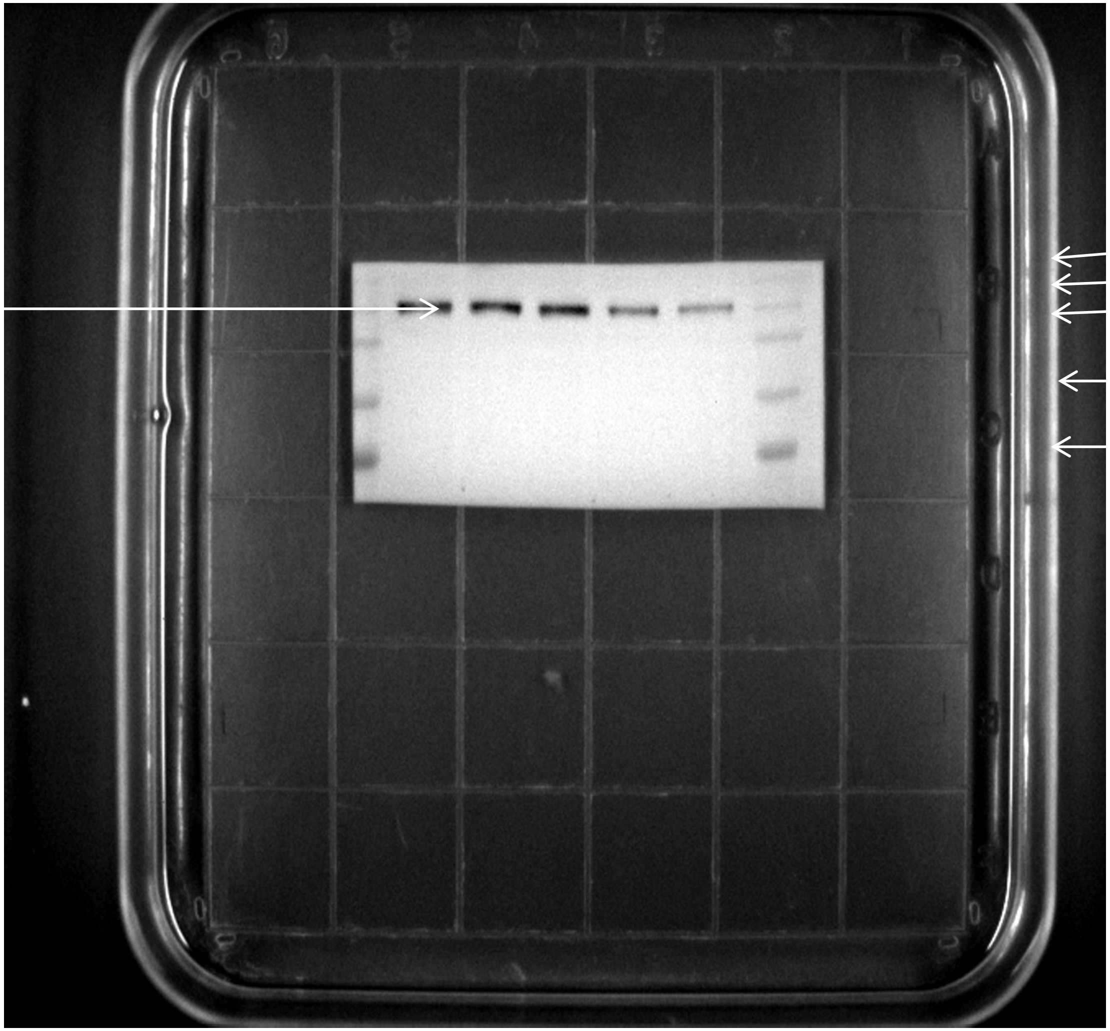

P-NLRC4, Invitrogen, MA5-31846, 1:1000; anti-Mouse IgG, Jackson, 115-035-003, 1:2000

Figure 4G

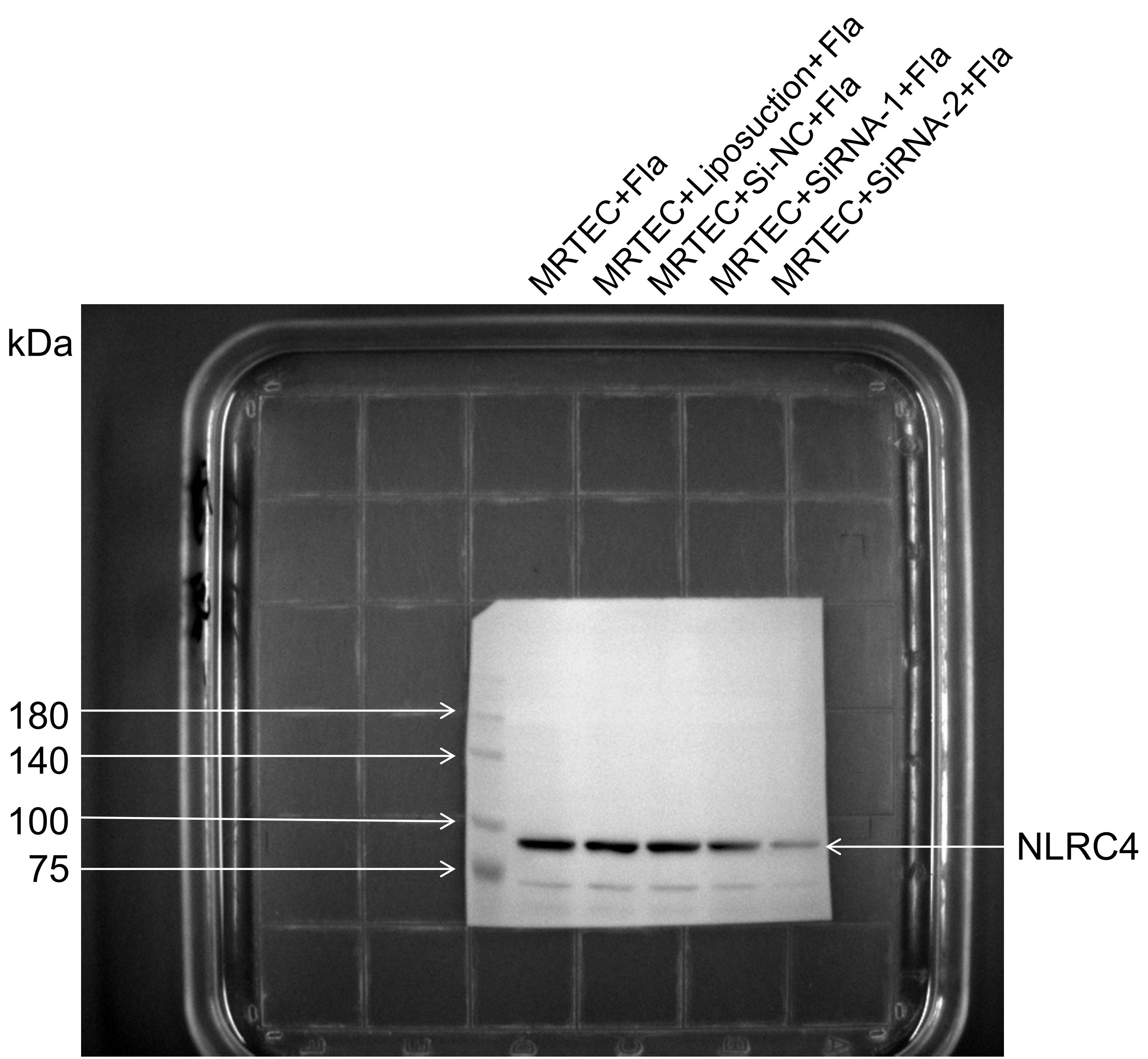

NLRC4, Invitrogen, PA5-88997, 1:1000; anti-Rabbit IgG, Jackson, 111-035-003, 1:2000

Figure 4G

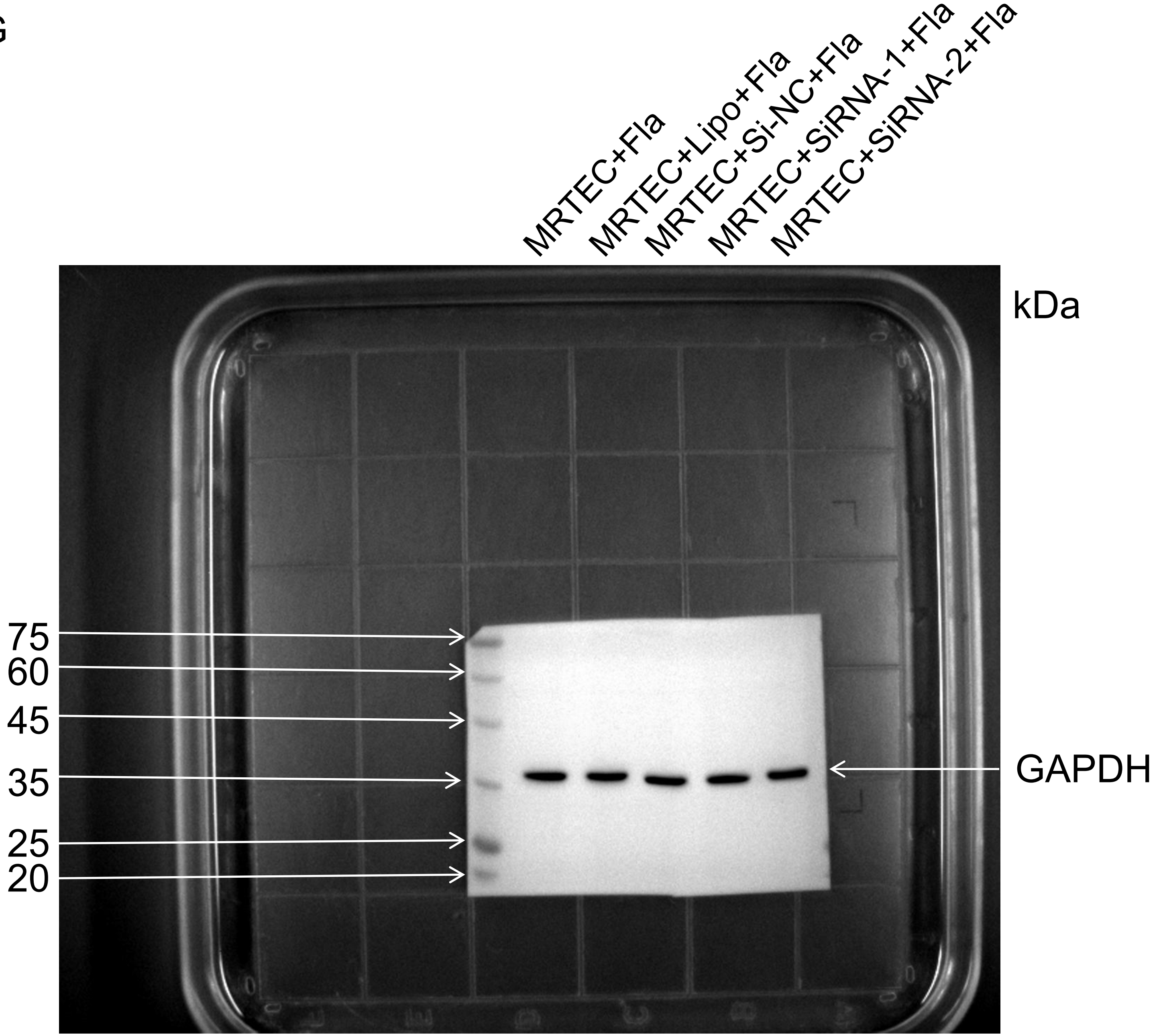

GAPDH, Proteintech, 60004-1-Ig, 1:10000;
anti-Mouse IgG, Jackson, 115-035-003, 1:5000

Figure 5E

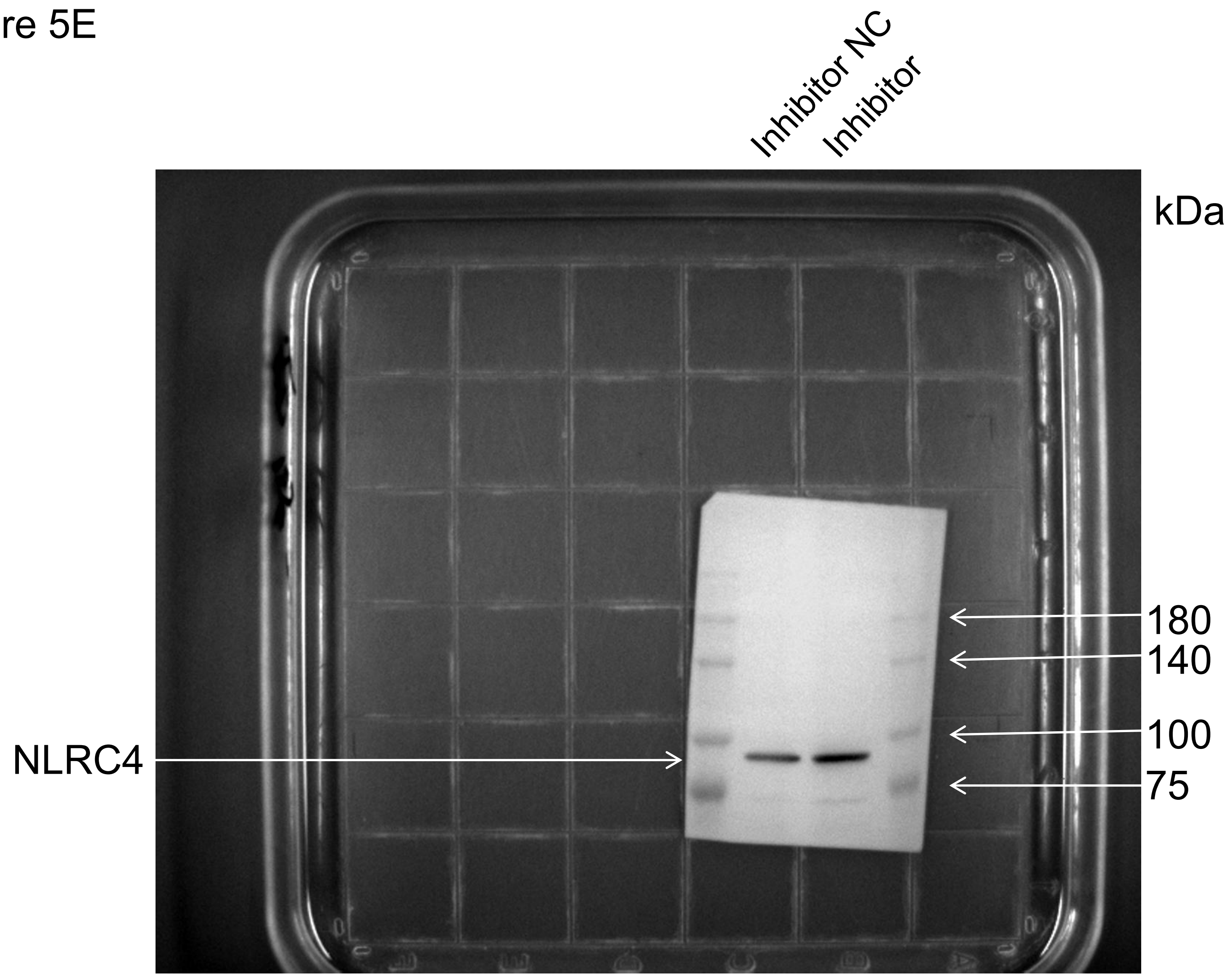

NLRC4, Invitrogen, PA5-88997, 1:1000;
anti-Rabbit IgG, Jackson, 111-035-003, 1:2000

Figure 5E

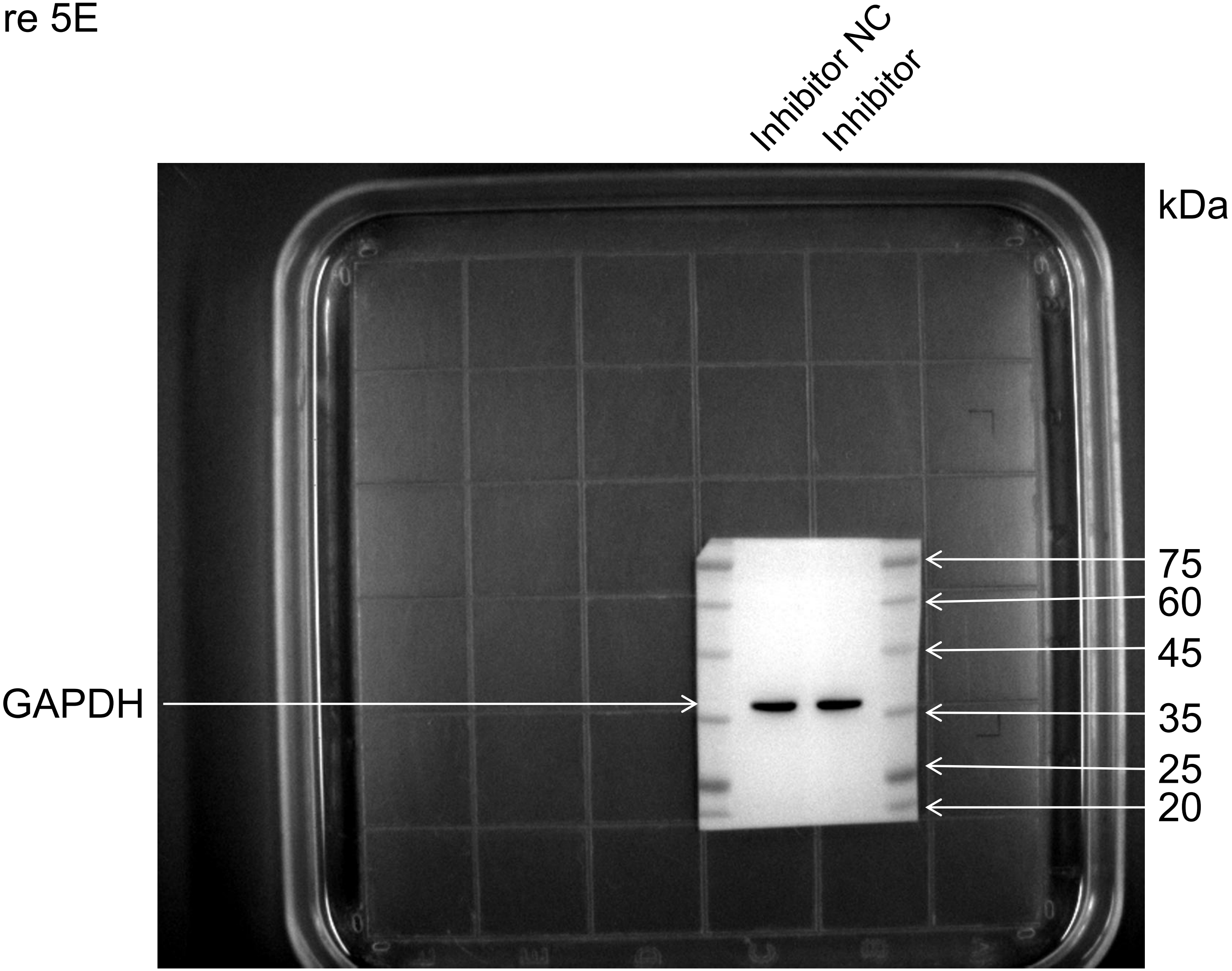

GAPDH, Proteintech, 60004-1-Ig, 1:10000;
anti-Mouse IgG, Jackson, 115-035-003, 1:5000

Figure 6A

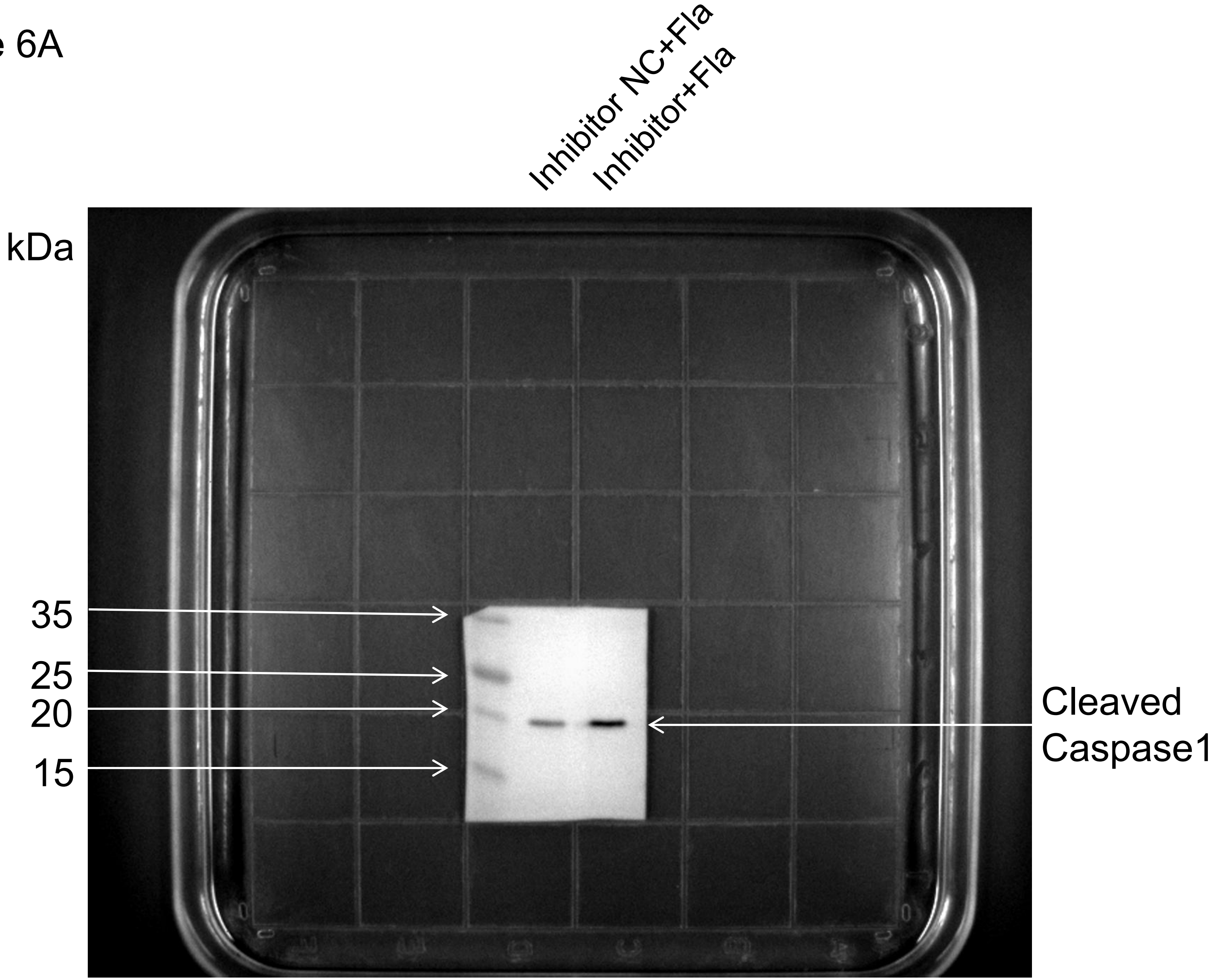

Cleaved Caspase1, AdipoGen, AG-20B-0042-C100, 1:1000, 20~50kD;
anti-Mouse IgG, Jackson, 115-035-003, 1:2000

Figure 6A

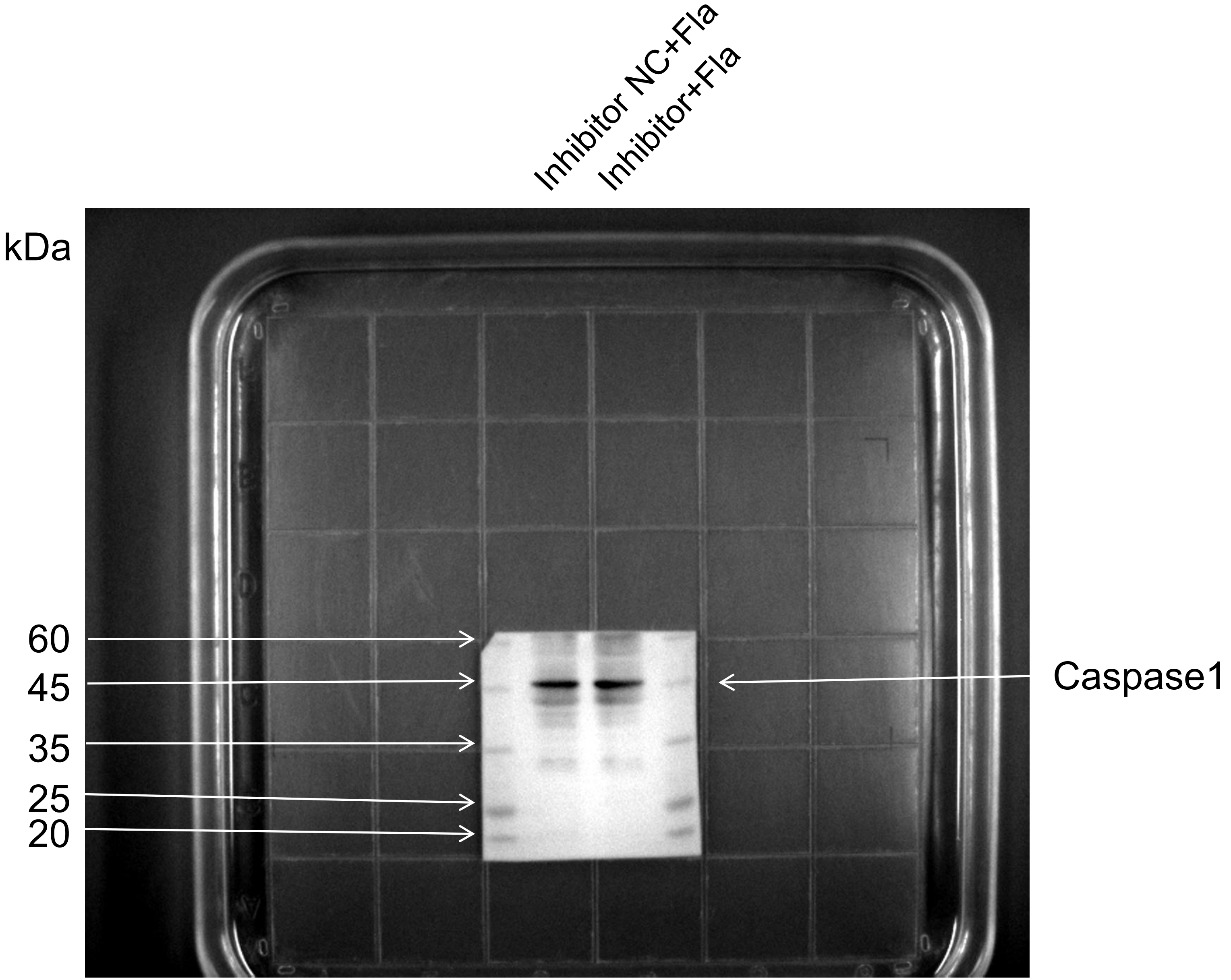

Caspase1, Proteintech, 22915-1-AP, 1:1000, 30/35/45~47kD;
anti-Rabbit IgG, Jackson, 111-035-003, 1:2000

Figure 6A

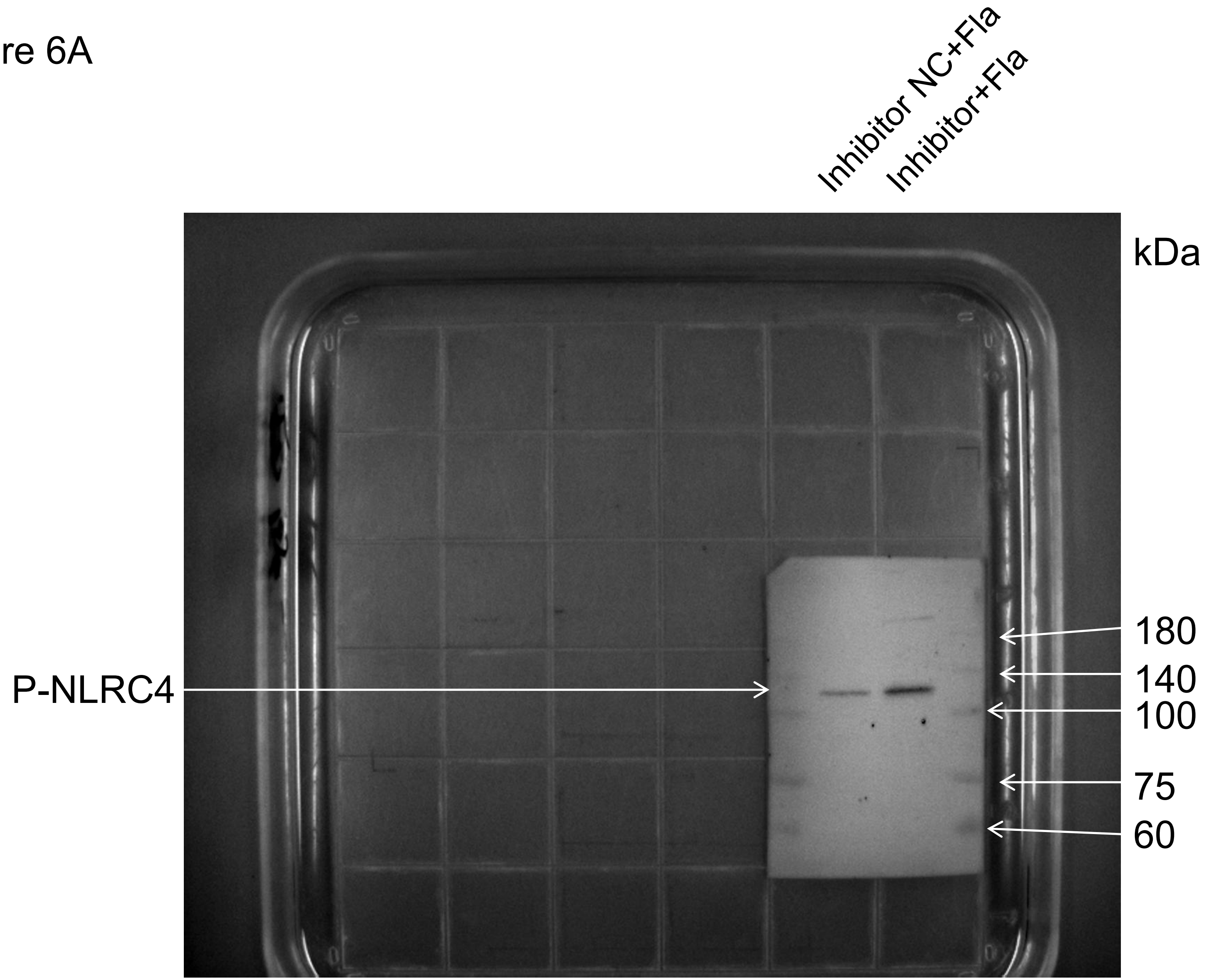

P-NLRC4, Invitrogen, MA5-31846, 1:1000;
anti-Mouse IgG, Jackson, 115-035-003, 1:2000

Figure 6A

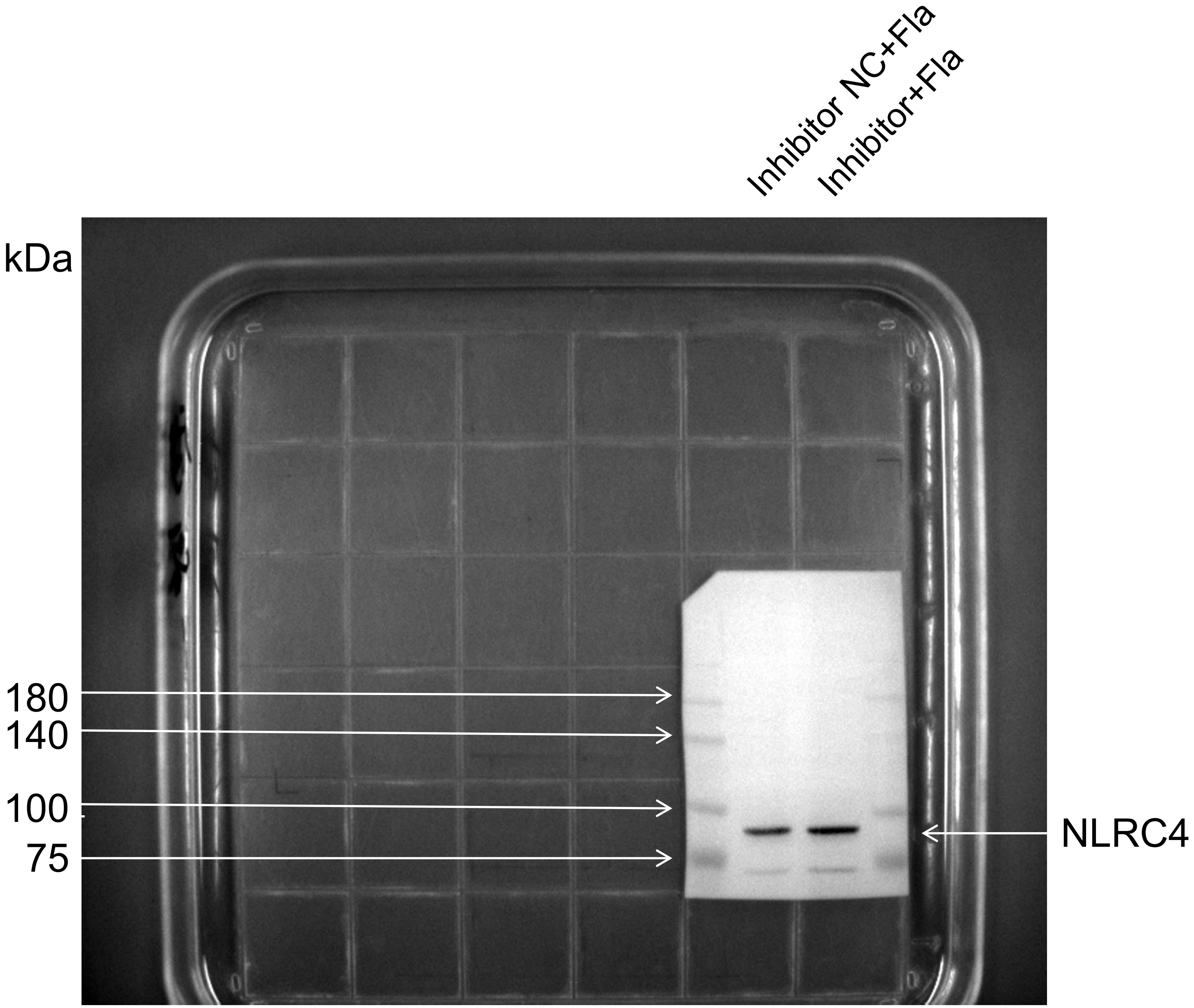

NLRC4, Invitrogen, PA5-88997, 1:1000;
anti-Rabbit IgG, Jackson, 111-035-003, 1:2000

Figure 6A

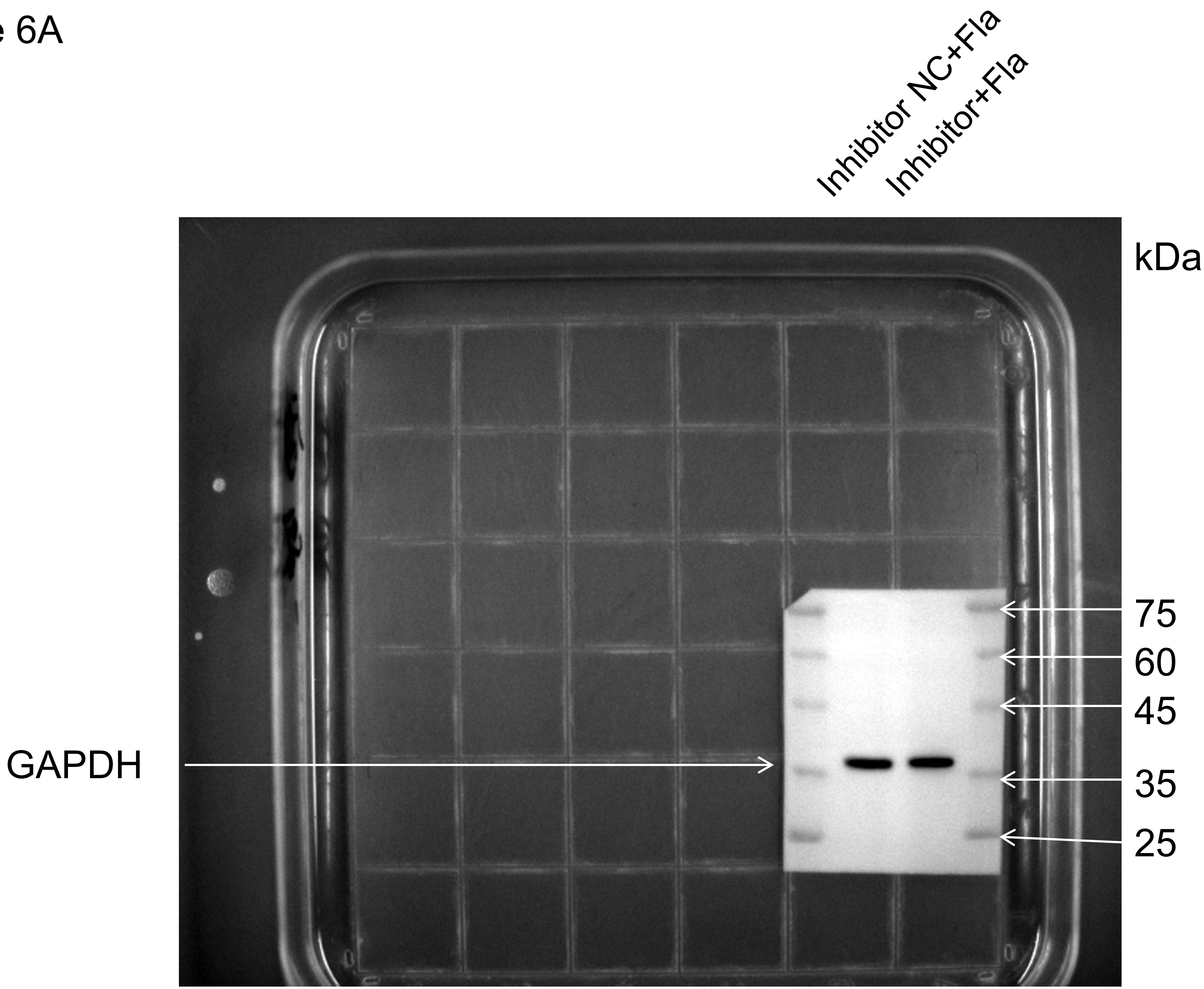

GAPDH, Proteintech, 60004-1-Ig, 1:10000;
anti-Mouse IgG, Jackson, 115-035-003, 1:5000

Figure 6D

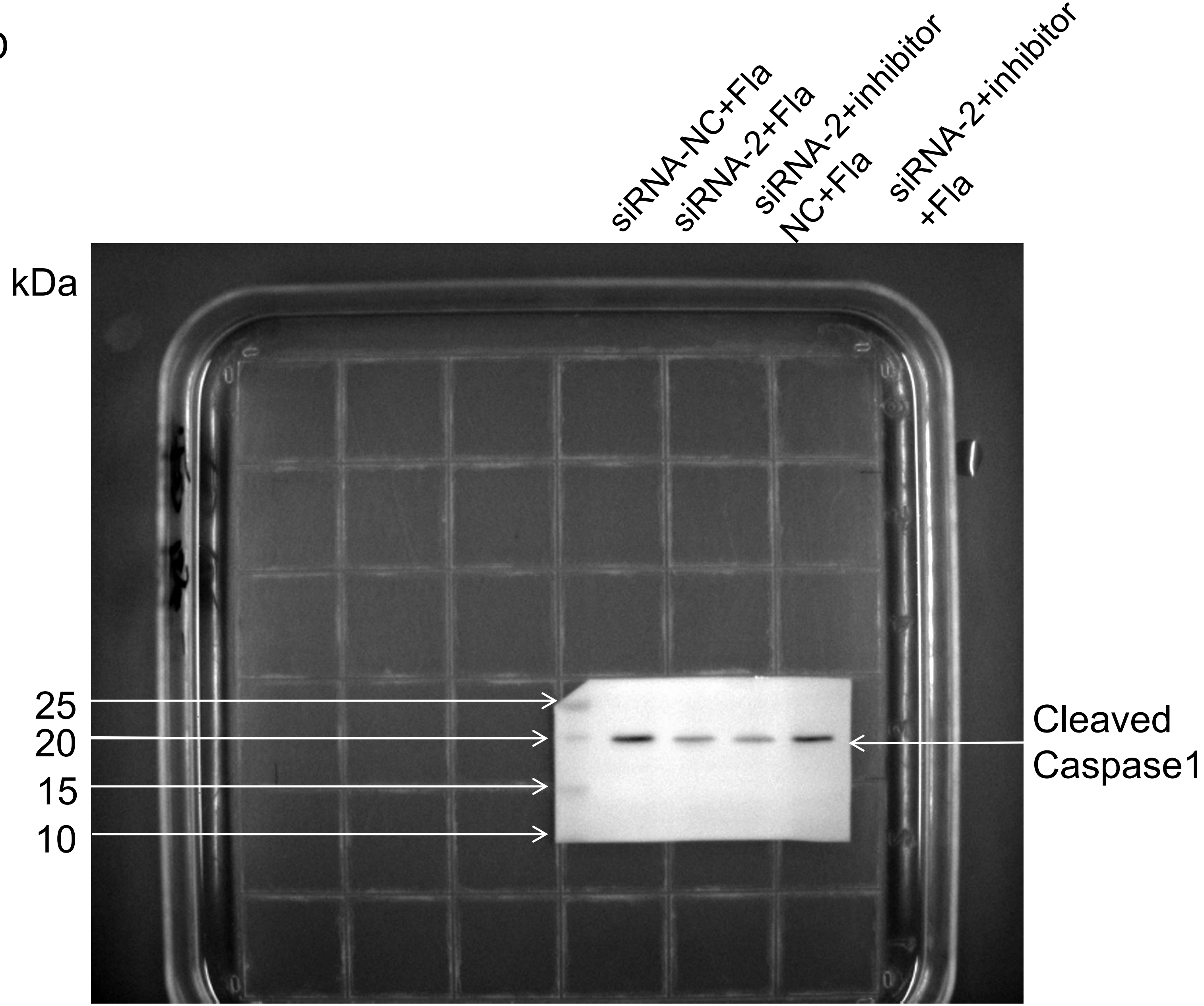

Cleaved Caspase1, AdipoGen, AG-20B-0042-C100, 1:1000, 20~50kD; anti-Mouse IgG, Jackson, 115-035-003, 1:2000

Figure 6D

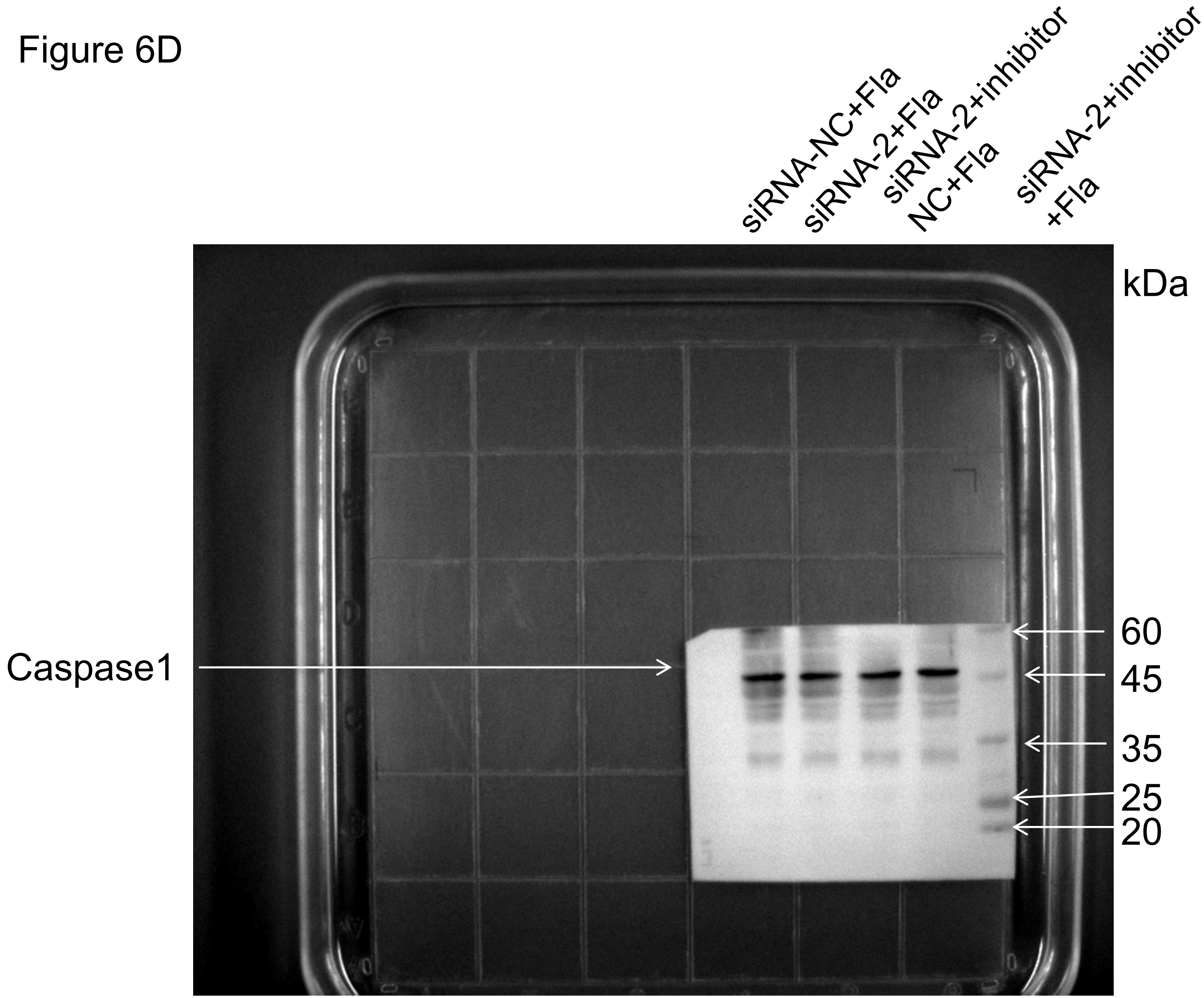

Caspase1, Proteintech, 22915-1-AP, 1:1000, 30/35/45~47kD;
anti-Rabbit IgG, Jackson, 111-035-003, 1:2000

Figure 6D

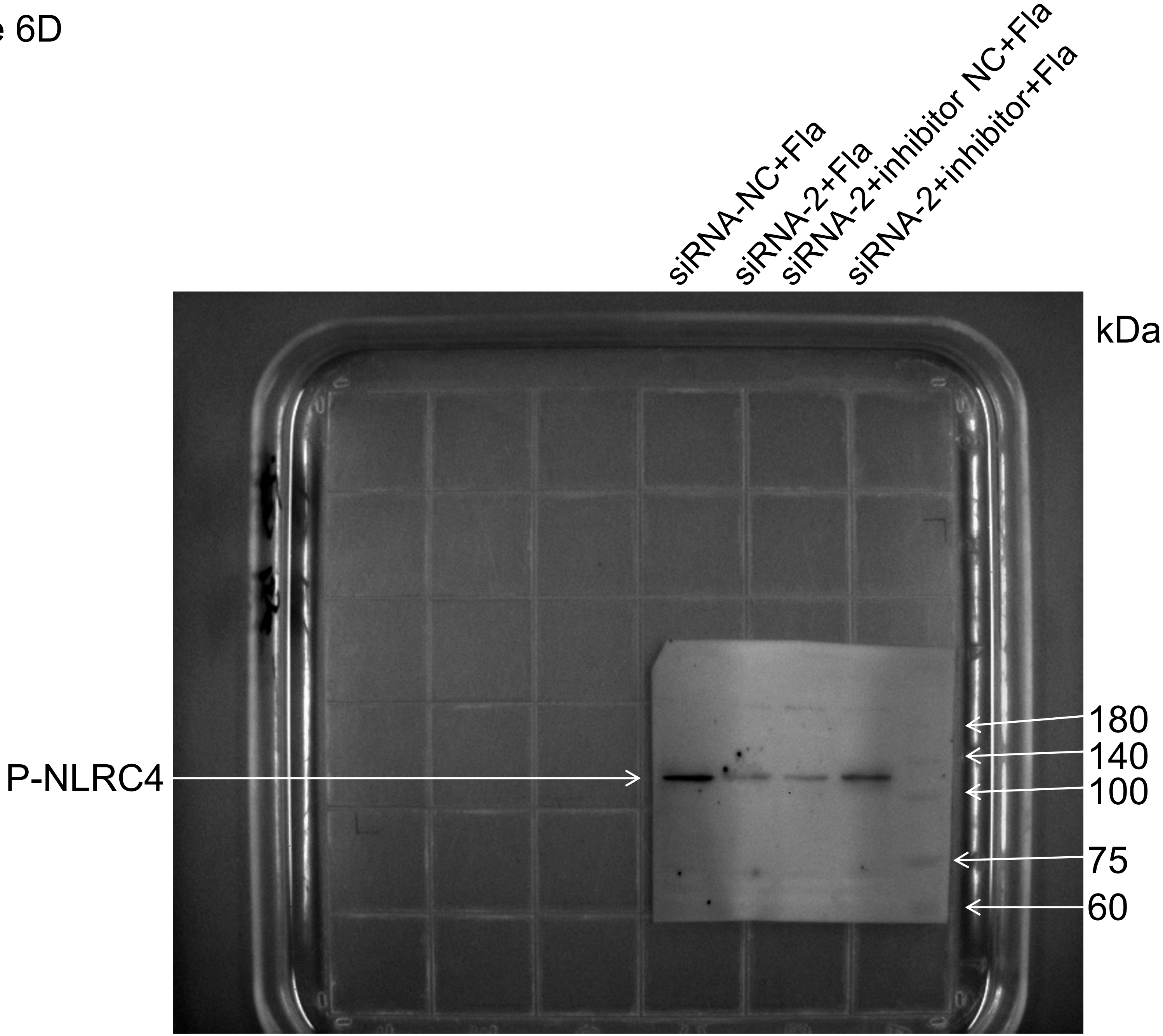

P-NLRC4, Invitrogen, MA5-31846, 1:1000; anti-Mouse IgG, Jackson, 115-035-003, 1:2000

Figure 6D

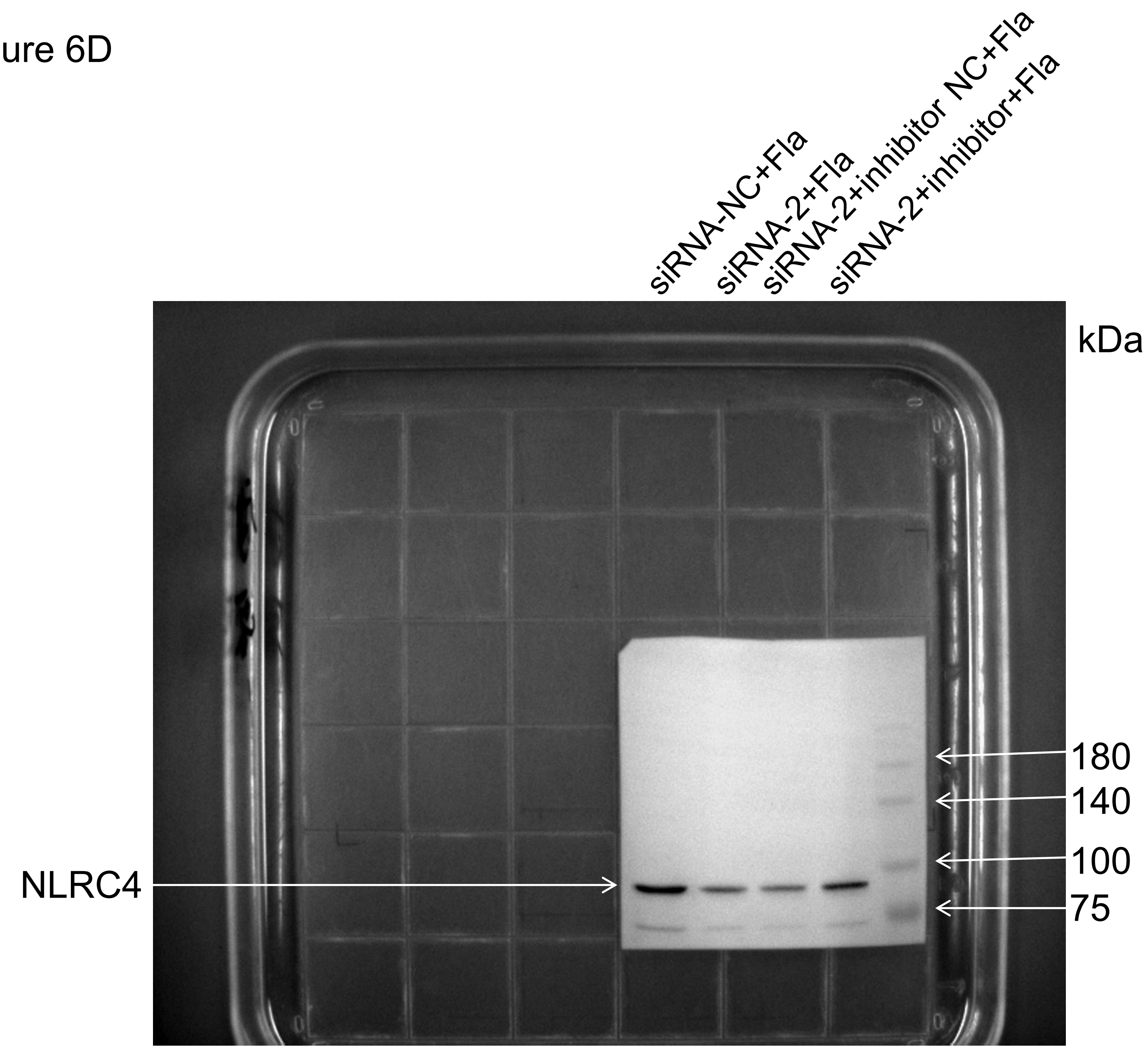

NLRC4, Invitrogen, PA5-88997, 1:1000; anti-Rabbit IgG, Jackson, 111-035-003, 1:2000

Figure 6D

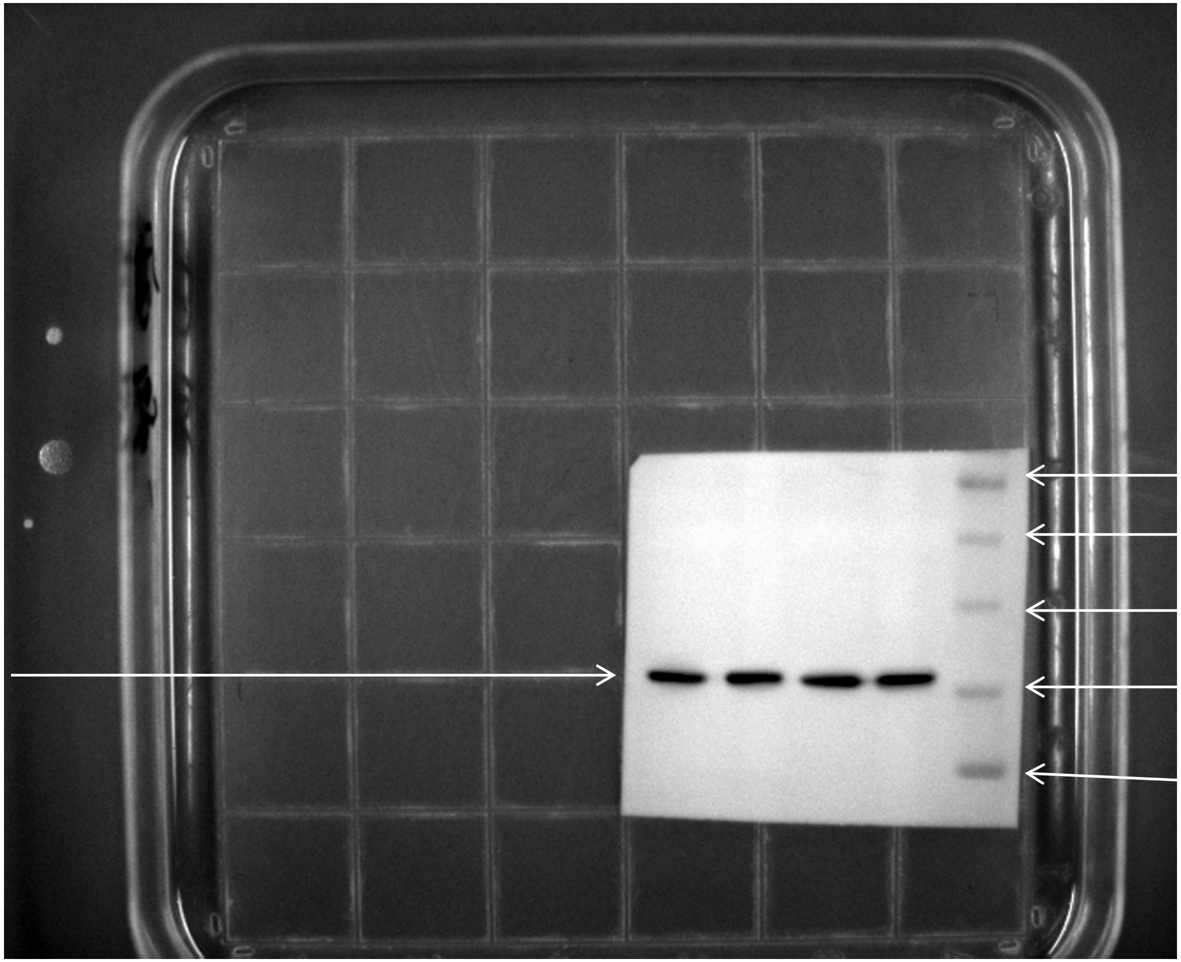

GAPDH,Proteintech, 60004-1-Ig,1:10000; anti-Mouse IgG, Jackson, 115-035-003,1:5000
