# Supplementary figures and images for: Circ_0000181 regulates miR-667-5p/NLRC4 axis to promote pyroptosis progression in diabetic nephropathy
Source: Sci Rep. 2022 Jul 14;12:11994. doi: 10.1038/s41598-022-15607-7 (PMC9283475; doi:10.1038/s41598-022-15607-7)

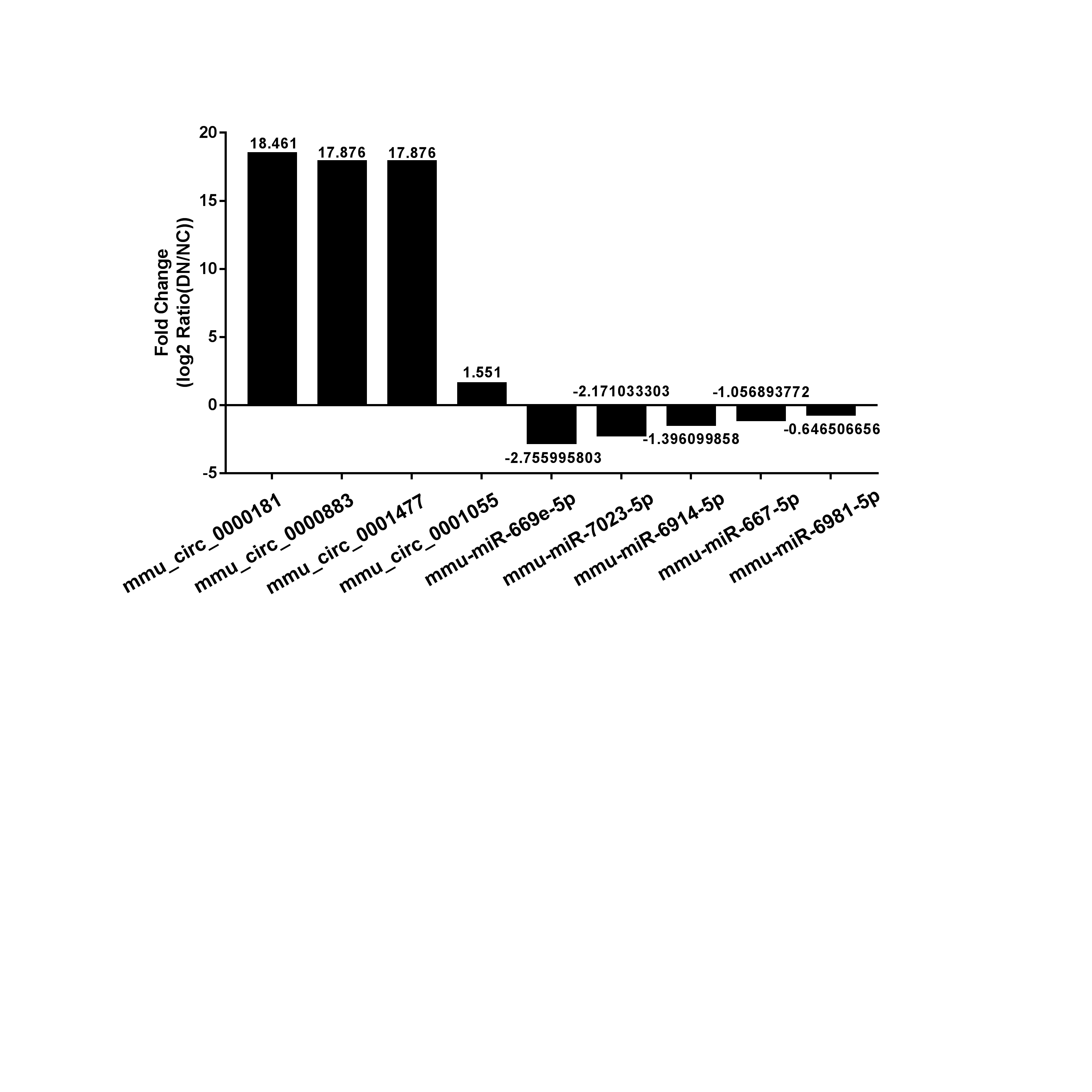

Supplement: Supplementary file 2 — Supplementary Figure S1. [file 41598_2022_15607_MOESM2_ESM.tif]
